# Surface-Specific Modification of Graphitic Carbon Nitride by Plasma for Enhanced Durability and Selectivity of Photocatalytic CO_2_ Reduction with a Supramolecular Photocatalyst

**DOI:** 10.1021/acsami.3c00955

**Published:** 2023-03-01

**Authors:** Noritaka Sakakibara, Mitsuhiko Shizuno, Tomoki Kanazawa, Kosaku Kato, Akira Yamakata, Shunsuke Nozawa, Tsuyohito Ito, Kazuo Terashima, Kazuhiko Maeda, Yusuke Tamaki, Osamu Ishitani

**Affiliations:** † Department of Chemistry, School of Science, Tokyo Institute of Technology, 2-12-1-NE-2 Ookayama, Meguro, Tokyo 152-8550, Japan; ‡ Japan Society for the Promotion of Science, Kojimachi Business Center Building, 5-3-1 Kojimachi, Chiyoda, Tokyo 102-0083, Japan; § Institute of Materials Structure Science, High Energy Accelerator Research Organization, Tsukuba, Ibaraki 305-0801, Japan; ∥ Faculty of Natural Science and Technology, Okayama University, 3-1-1, Tsushima-naka, Kita-ku, Okayama 700-8530, Japan; ⊥ Department of Advanced Materials Science, Graduate School of Frontier Sciences, The University of Tokyo, 5-1-5 Kashiwanoha, Kashiwa, Chiba 277-8561, Japan; # Department of Chemistry, Graduate School of Advanced Science and Engineering, Hiroshima University, 1-3-1 Kagamiyama, Higashi-Hiroshima, Hiroshima 739-8526, Japan

**Keywords:** photocatalysis, CO_2_ reduction, carbon nitride, metal complex, plasma

## Abstract

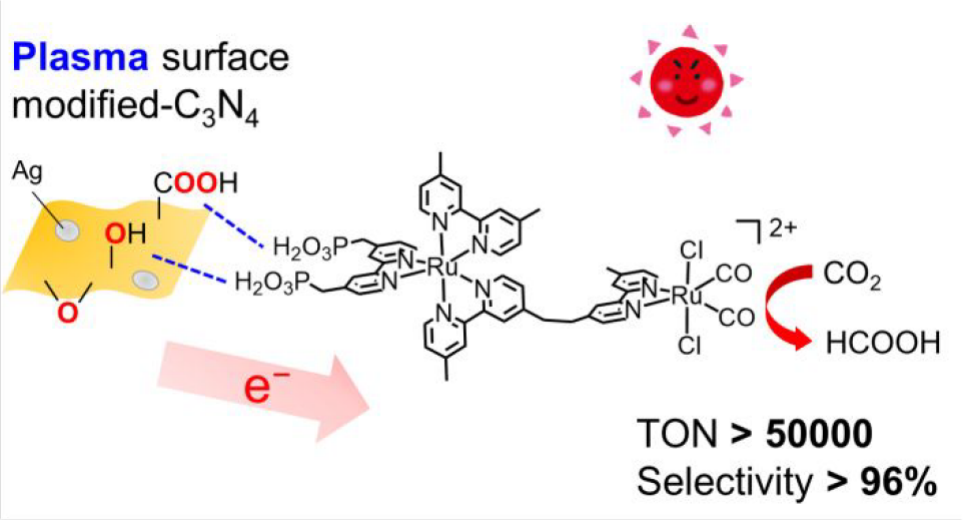

Photocatalytic CO_2_ reduction is in high demand for sustainable energy management. Hybrid photocatalysts combining semiconductors with supramolecular photocatalysts represent a powerful strategy for constructing visible-light-driven CO_2_ reduction systems with strong oxidation power. Here, we demonstrate the novel effects of plasma surface modification of graphitic carbon nitride (C_3_N_4_), which is an organic semiconductor, to achieve better affinity and electron transfer at the interface of a hybrid photocatalyst consisting of C_3_N_4_ and a Ru(II)–Ru(II) binuclear complex (**RuRu′**). This plasma treatment enabled the “surface-specific” introduction of oxygen functional groups via the formation of a carbon layer, which worked as active sites for adsorbing metal-complex molecules with methyl phosphonic-acid anchoring groups onto the plasma-modified surface of C_3_N_4_. Upon photocatalytic CO_2_ reduction with the hybrid under visible-light irradiation, the plasma-surface-modified C_3_N_4_ with **RuRu′** enhanced the durability of HCOOH production by three times compared to that achieved when using a nonmodified system. The high selectivity of HCOOH production against byproduct evolution (H_2_ and CO) was improved, and the turnover number of HCOOH production based on the **RuRu′** used reached 50 000, which is the highest among the metal-complex/semiconductor hybrid systems reported thus far. The improved activity is mainly attributed to the promotion of electron transfer from C_3_N_4_ to **RuRu′** under light irradiation via the accumulation of electrons trapped in deep defect sites on the plasma-modified surface of C_3_N_4_.

## Introduction

Owing to the rising importance of sustainable and carbon-neutral energy management, effective conversion of CO_2_ into chemical feedstocks with high energy content is highly desirable. Among the various strategies for CO_2_ conversion,^1−5^ photocatalysis is a promising approach, as photocatalysts enable the multielectron reduction of CO_2_ via the harvesting of solar energy to produce energy-rich materials.^6−10^ Metal complexes,^6,7^ inorganic semiconductors,^10^ and their hybrid photocatalysts^8,9^ have been vigorously developed as effective strategies for CO_2_ utilization.

In recent years, organic semiconductors,^11^ such as graphitic carbon nitride (C_3_N_4_),^12−14^ conjugated polymers (CPs),^15−18^ and covalent organic frameworks (COFs),^19−21^ have emerged as a new class of active photocatalysts; the photophysical and electronic properties of organic semiconductors can be rationally managed by designing building blocks by adjusting the monomer structures. In particular, C_3_N_4_ is a pioneering visible-light-responsive organic semiconductor photocatalyst that has been highly studied since Wang and co-workers reported photocatalytic H_2_ production by C_3_N_4_ with a platinum cocatalyst under visible light irradiation with triethanolamine as a sacrificial electron donor;^12^ it can be utilized for various photon-energy conversion applications. Although various attractive designability and photoactive properties of organic semiconductors have been reported, selective photocatalytic reduction of CO_2_ remains a challenge.^18^

**Chart 1:**
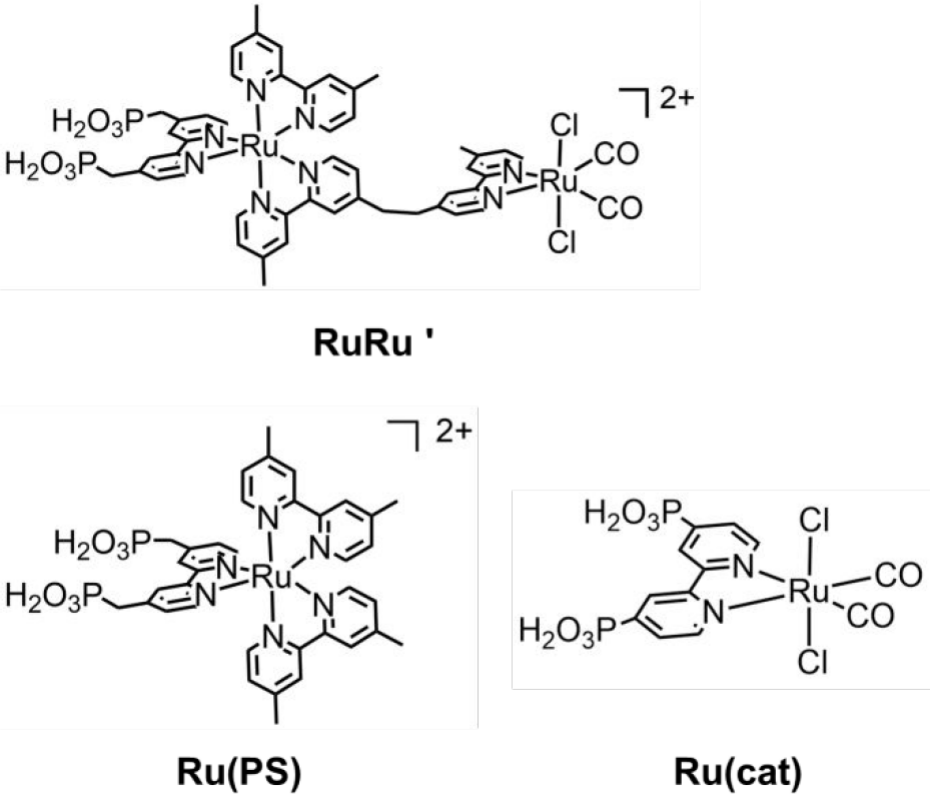
Structures and Abbreviations of **RuRu′** and Its Model Mononuclear Complexes

Metal-complex photocatalysts are highly advantageous for enhancing the selectivity of CO_2_ reduction.^6,22−24^ In particular, supramolecular photocatalysts, which are composed of two metal complexes as the photosensitizer and catalyst and are directly connected with a bridging ligand, have accomplished efficient photocatalytic CO_2_ reduction with high selectivity and durability under visible light irradiation.^7,22^ For example, ruthenium(II)–rhenium(I)^25^ and binuclear ruthenium(II)^26,27^ complexes (**RuRu′** in Chart 1 as an example), in which an ethylene chain connects two metal complexes, have been reported as visible-light-responsive supramolecular photocatalysts for CO_2_ reduction with high durability and high formation selectivity of CO or HCOOH.

**Scheme 1:**
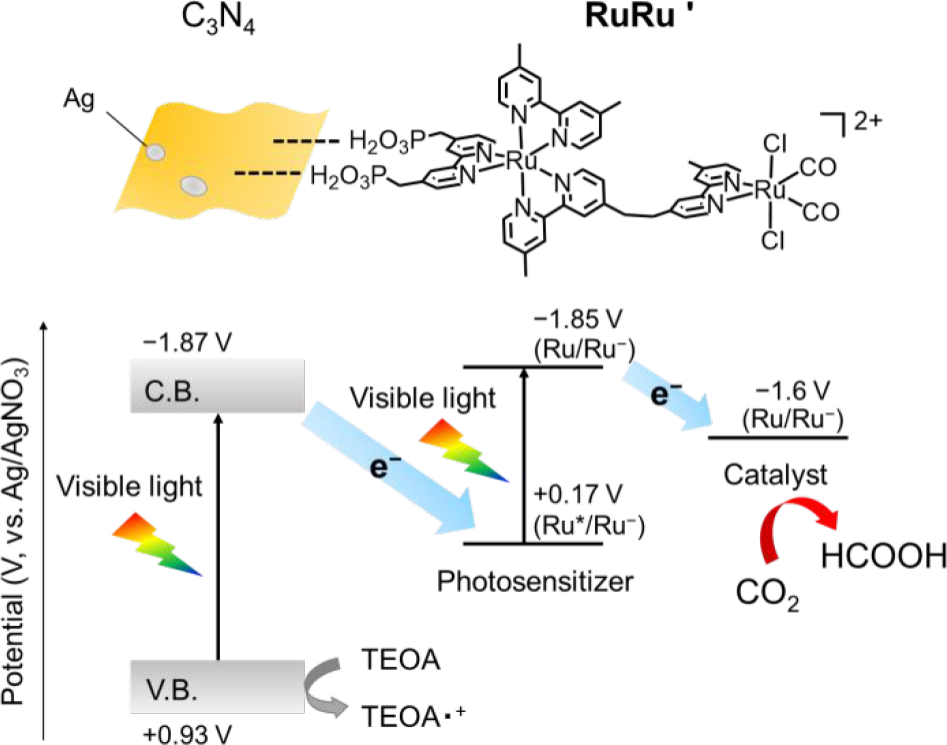
Energy Diagram of CO_2_ Reduction by the Z-Scheme Hybrid Photocatalyst under Visible Light Irradiation

Recently, hybrid photocatalysts combining such supramolecular photocatalysts with semiconductors have been developed, which can ensure both strong reduction and oxidation powers owing to a Z-scheme type electron transfer involving the step-by-step excitation of the semiconductor and photosensitizer units in the supramolecular photocatalyst.^8,28−33^ In such hybrid photocatalysts, the deposition of Ag particles on the semiconductors enhances the photocatalysis of the hybrid systems; this is mainly owing to an increase in efficiencies of electron–hole separation during excitation in the semiconductors and electron transfer from the semiconductors to the supramolecular photocatalysts.^8^ By assembling Ag loaded-C_3_N_4_ (Ag/C_3_N_4_) and binuclear ruthenium(II) complexes as shown in Scheme 1, for example, highly selective formic acid (HCOOH) production with very high durability (TON > 30 000 based on the supramolecular used) has been accomplished under visible-light irradiation.^28,29^

In these hybrid photocatalysts, the design of the interface between the semiconductor and supramolecular photocatalyst is indispensable for improving the photocatalytic CO_2_ reduction activity.^8,32^ For example, manipulation of the C_3_N_4_ nanosheet surface by depositing TiO_2_ has been reported for improving the separation of photoexcited charge carriers and for increasing the adsorption sites of the supramolecular photocatalyst with phosphonic-acid anchoring groups, which induced an increase in the photocatalysis for CO_2_ reduction.^31^ In principle, high adsorptivity of the supramolecular photocatalyst is crucial for improving the efficiency and durability of supramolecular photocatalysts on solid materials.^34^ However, C_3_N_4_ itself has amino groups only at the edges of its sheet structure,^35^ which limits the number of adsorption sites for the supramolecular photocatalyst. Inorganic semiconductors (e.g., metal oxides and metal oxynitride) can adsorb many molecules with phosphonic-acid anchoring groups, typically with a surface coverage of over 70%.^36,37^ Meanwhile, C_3_N_4_ adsorbs such molecules with only ∼20% surface coverage.^31^ The limited number of adsorption sites on C_3_N_4_ hinders further improvement of the interfacial manipulation of the hybrid photocatalyst. This should be also the case for other types of organic semiconductors, except for the intentional introduction of functional groups into building blocks.^38^

To gain abundant adsorption sites on the surface of C_3_N_4_, plasma surface modification should have great potential; this is an attractive technique that modifies and improves the surface properties of organic polymers by forming functional groups (e.g., hydroxyl, carboxyl, and amino functional groups).^39−42^ One noticeable advantage of plasma is its nonequilibrium reaction field; that is, a high electron temperature in the 1–10 eV range can provide reactive radical species while keeping the ambient temperature low (≤room temperature).^43,44^ The plasma-modified surface can enhance the affinity with other targeted molecules.^39,45^ However, reports on the plasma treatments of C_3_N_4_^46−49^ and other organic molecules^50^ as photocatalysts have thus far not reported the improvement of the adsorptivity of photocatalyst molecules on organic semiconductors in hybrid photocatalysts. In previous reports on the plasma surface modification of C_3_N_4_, plasmas were generated in the gas phase (typically Ar gas was used), with O_2_, N_2_, or NH_3_ used as feeding gases to introduce oxygen- or nitrogen-containing functional groups and/or surface defect sites on C_3_N_4_,^46−50^ resulting in the direct modification of the C_3_N_4_ surface. These previous reports only focused on the improvement of charge separation of photoexcited carriers and/or dispersibility of C_3_N_4_ particles in solvents. However, when considering the photocatalytic CO_2_ reduction with hybrid photocatalysts of C_3_N_4_ and supramolecular photocatalysts, the direct surface modification of C_3_N_4_ as a CO_2_ reduction photocatalyst might cause undesirable side reactions, such as photocatalytic H_2_ production at the plasma-modified sites on C_3_N_4_.

By contrast, our group recently reported the plasma surface modification of hexagonal boron nitride nanosheets^51^ and multiwalled carbon nanotubes^52^ using plasma generated in an aqueous solution containing hydroquinone as a precursor for oxidative surface modification. It was reported that in this plasma surface modification, polymerization of the added hydroquinone enabled carbon layer deposition on the particles.^52,53^ We apply this method to C_3_N_4_ to form an additional carbon layer on the surface of C_3_N_4_ via oxidative surface modification of the π–π stacked hydroquinone precursor, which might suppress the possible side reactions at the plasma-modified sites on C_3_N_4_. Furthermore, the plasma generated in aqueous solution is advantageous for the introduction of functional groups at a high density, as the functional groups can be introduced not only at the edges, but also onto the in-planes of nanosheet powders.^54^ Therefore, the plasma generated in aqueous solution containing hydroquinone is potentially suitable for improving the photocatalysis of hybrid photocatalysts consisting of supramolecular photocatalysts and C_3_N_4_.

Herein, we report the plasma surface modification of C_3_N_4_ nanosheets using plasma generated in an aqueous solution containing hydroquinone as a precursor. The modification was intended to improve the interfacial affinity in a Z-scheme hybrid photocatalyst composed of C_3_N_4_ and a supramolecular photocatalyst **RuRu′**, which has a Ru(II) photosensitizer unit with methyl phosphonic acid anchoring groups and a Ru(II) catalyst unit (Chart 1 and Scheme 1). Among several types of C_3_N_4_ morphologies (e.g., bulk,^12^ mesoporous,^28^ and nanosheet^29^), nanosheet-type C_3_N_4_ was chosen in this study as it contained enough space for the attachment of the supramolecular photocatalyst even after the formation of the carbon layer through plasma treatment. Plasma treatment accomplished “surface-specific” modification of C_3_N_4_ with the formation of an oxygen-rich carbon layer on its surface, which significantly increased the number of adsorption sites of **RuRu′** molecules. Upon photocatalytic CO_2_ reduction by this hybrid photocatalyst, the plasma surface modification of C_3_N_4_ noticeably improved the photocatalysis compared to the nontreated hybrid photocatalyst: a 3-fold enhancement in the turnover number for HCOOH production while maintaining over 96% product selectivity. Under optimal conditions, the turnover number for HCOOH production was over 50 000, which is the highest among reported metal-complex/semiconductor hybrid systems for photocatalytic CO_2_ reduction.^28^ The mechanism of these positive effects of the plasma surface modification on the photocatalysis was investigated using time-resolved infrared absorption spectroscopy and electron spin resonance measurements.

## Materials and Methods

### Materials

Metal complexes used in this work (i.e., **RuRu′**, **Ru(PS)**, and **Ru(cat)**) were synthesized according to previously reported methods.^7,55−57^*N,N*-dimethylacetamide (DMA), triethanolamine (TEOA), and acetonitrile (MeCN) were distilled and stored under Ar prior to use. H_2_O was distilled and deionized. ^13^CO_2_ (99% ^13^C) was purchased from Cambridge Isotope Laboratories, Inc. Tetrabutylammonium tetrafluoroborate (Et_4_NBF_4_) was recrystallized from MeCN/ethyl acetate and then dried under a vacuum at 373 K overnight before utilization. All other reagents were commercially available and used without further purification.

### Synthesis and Plasma Treatment of C_3_N_4_

C_3_N_4_ nanosheets were synthesized using a previously reported procedure by heating 10 g of urea (>99%, Wako Pure Chemicals Co.) at 823 K in air for 2 h in a crucible in a muffle furnace.^29^ Plasma treatment of C_3_N_4_ was conducted in a hydroquinone-containing aqueous solution using a previously reported setup.^52,53^ Nonequilibrium plasma was generated in a NaCl solution (0.1 g L^−1^) containing 1 wt % C_3_N_4_ and 4 wt % hydroquinone (>99%, Wako Pure Chemicals Co.) in 100 mL of deionized water and dispersed using a magnetic stirrer. A bipolar pulse with an amplitude of 1.6 kV, a current of 6 A, a repetition frequency of 80 kHz, and a pulse width of 0.4 ms was applied to tungsten rods (diameter: 1 mm) separated by a 2 mm gap, and the plasma was maintained for 1 h. Figure S1 shows the voltage–current characteristics, optical emission spectrum, and a photograph of the plasma. After plasma treatment, the remaining hydroquinone and byproducts were removed using water, methanol, DMA, and acetone, and the washed plasma-C_3_N_4_ was dried overnight under a vacuum.

### Ag or Pt Loading on C_3_N_4_

Ag nanoparticles were loaded at 1.25 wt % on C_3_N_4_ by the impregnation method with AgNO_3_ (>99.8%, Wako Pure Chemicals Co.) as a precursor. Subsequently, 100 mg of C_3_N_4_ was dispersed in 10 mL of H_2_O, followed by the addition of an aqueous AgNO_3_ solution. The dispersed solution was distilled under reduced pressure to remove the H_2_O. The resulting solid sample was heated under a H_2_ stream (20 mL min^–1^) at 473 K for 1 h. Platinum (Pt) nanoparticles were loaded at 5 wt % on C_3_N_4_ by using the photodeposition method. 40 mg of C_3_N_4_ was dispersed in an aqueous solution of H_2_PtCl_6_ (>99.5%, Wako Pure Chemicals Co.) with 10 vol% methanol as a sacrificial electron donor and irradiated with a Xe lamp (λ > 350 nm) for 4 h.

### Adsorption and Desorption Experiments of **RuRu′** on C_3_N_4_

The C_3_N_4_ or plasma-C_3_N_4_ was dispersed in a MeCN solution of **RuRu′**, and the suspension was stirred in the dark at room temperature for 5 days to allow for the adsorption/desorption equilibrium. The obtained powder was collected via filtration, washed with MeCN, and dried under a vacuum. The absorbance of the filtrate was measured using a V-570 spectrometer (JASCO). The amount (AA) and average density (AD) of adsorbed metal complexes were calculated using the following equations:
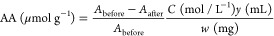
1
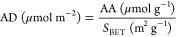
2where *A*_before_ and *A*_after_ are the absorbances at 460 nm (corresponding to ^1^MLCT absorption) of **RuRu′** before and after the adsorption procedure, respectively; *C* is the initial concentration of **RuRu′**; *y* is the amount of **RuRu′** solution; and *w* is the weight of C_3_N_4_, which was added for the adsorption. The adsorption density is a spatially averaged value, assuming that **RuRu′** is uniformly distributed on C_3_N_4_.

The stability of adsorption was evaluated by the desorption of **RuRu′** from C_3_N_4_. Four milligrams of **RuRu′**-adsorbed C_3_N_4_ (**RuRu′**/C_3_N_4_) was dispersed in 4 mL of DMA/TEOA (4:1, v:v) and stirred in the dark at room temperature for 5 days. The desorption rate of **RuRu′** was estimated from the absorbance of the filtrate using the following equation:
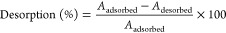
3where *A*_adsorbed_ is the absorbance of the **RuRu′** solution at 460 nm with a concentration (μM) of AA (μmol g^–1^) × 4 (mg)/4 (mL), which corresponds to the amount of adsorbed **RuRu′** before desorption, and *A*_adsorbed_ is the absorbance of the filtrate at 460 nm after desorption.

The other metal complexes were hybridized with C_3_N_4_, plasma-C_3_N_4_, Ag/C_3_N_4_, or Ag/plasma-C_3_N_4_ in the same manner as in the adsorption experiment described above.

### Characterization of Powders

XRD patterns were recorded using a MiniFlex600 (Rigaku) powder diffractometer. FT-IR spectra were obtained using an FT/IR-6700 (JASCO) spectrometer with an ATR configuration. Diffuse reflectance absorption spectra were obtained by utilizing a V-670 (JASCO) spectrometer equipped with an integration sphere using a Spectralon reference standard (6916-H422A, JASCO) as a reference. TG measurements (DTG-60, Shimadzu) were performed under a flow of air (90 mL min^–1^) at a ramp rate of 5 K min^–1^. The BET surface areas were measured using a BELSORP Max-II (MicrotracBEL) at liquid N_2_ temperature (77 K). Prior to adsorption–desorption measurements, the samples were heated at 150 °C in vacuum for 720 min. The surface atomic compositions were examined using X-ray photoelectron spectroscopy (XPS; ESCA-3400, Shimadzu). XPS data were corrected using the O 1s peak (532.6 eV) of the adsorbed water molecules as an internal reference. The backgrounds of the spectra were subtracted using the Shirley method. The decomposition of the C 1s spectra was performed using a Gaussian function while maintaining the peak position of each component between the as-C_3_N_4_ and plasma-C_3_N_4_. The ratio of oxygen-containing groups was estimated by dividing the sum of the integrals of the decomposed C–O and C—O curves by the total integral of the spectrum. The ICP-OES measurement was performed (5100 VDV ICP-OES, Agilent Technologies) for quantification of the amount of Ru (ions) on **RuRu′**/Ag/C_3_N_4_ by using 10 mL of a nitric acid solution in which 4 mg of **RuRu′** on **RuRu′**/Ag/C_3_N_4_ was dissolved.

### XAFS Measurement

X-ray absorption fine structure (XAFS) measurements of the Ag–K edge spectra of **RuRu′**/Ag/C_3_N_4_ were carried out using the AR-NW10A beamline of the High Energy Accelerator Research Organization (PF-AR, Tsukuba, Japan). XAFS spectra were acquired at room temperature in transmittance mode using a Si(311) double-crystal monochromator. The data for the XAFS spectra were processed using Athena.^58^ Fourier transforms of the *k*^3^-weighted EXAFS spectra were typically in the 3.0–12.0 Å region.

### Photocatalysis

In photocatalytic CO_2_ reduction experiments, the prepared photocatalyst particles were dispersed in an 8 mL glass test tube, which was filled with 4 mL of a DMA/TEOA mixed solution with a 4:1 volume ratio. Prior to irradiation, the suspension was purged with CO_2_ for 30 min. Visible light was irradiated by stirring using a merry-go-round-type photoirradiation apparatus, Iris-MG (CELL System Co.), equipped with 410 and 460 nm LED light sources. The output powers of the LEDs were 4 mW and 17.5 mW at 410 and 460 nm, respectively. Figure S7 shows the spectrum of the irradiated light. Gaseous products of photocatalysis, namely, CO and H_2_, were analyzed using a gas chromatograph with a thermal conductivity detector (GC-TCD) (GL Science GC323), an activated carbon column, and argon carrier gas. Formic acid (HCOOH) generated in the liquid phase was analyzed using a capillary electrophoresis system (Agilent Technologies 7100 L).

In the photocatalytic H_2_ production experiments, 4 mg of photocatalyst powder was dispersed in an 8 mL glass test tube, which was filled with 4 mL of a H_2_O/TEOA mixed solution with a 4:1 volume ratio. The procedures for light irradiation and product characterization were the same as those described above for the photocatalytic CO_2_ reduction.

The apparent quantum yields (AQYs) for HCOOH production during photocatalytic CO_2_ reduction were determined using a 300 W xenon lamp (MAX-303, Asahi Spectra) with a band-pass filter of 400 nm. AQYs were estimated using the following equation:
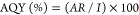
4where *R* and *I* represent the numbers of HCOOH and incident photons, respectively, and *A* = 4 indicates the coefficient of reaction. The total number of incident photons (10 mW) was measured using a spectroradiometer (Eko Instruments, LS-100).

### Isotope Tracer Experiment

^13^CO_2_ gas was introduced into a DMA/TEOA mixed solution (4:1 (v/v), 4.0 mL) containing 4.0 mg of the photocatalyst powder after the solution underwent freeze–pump–thaw degassing. The ^1^H NMR spectra of the reaction solutions were obtained using a JNM-ECA 400 spectrometer (JEOL) using the No-D technique. The solids were removed by filtration before the measurements were performed.

### Time-Resolved Infrared Absorption Spectroscopy

Measurements were performed using a homemade spectrometer.^59^ C_3_N_4_ powders before and after the plasma treatment were deposited onto a CaF_2_ plate at a density of 0.75 mg cm^–2^ and placed in a vacuum cell. The cells were then purged with N_2_ gas. The samples were photoexcited by a 420 nm visible pulse from an Nd:YAG laser (Continuum Surelite II; pulse duration = 6 ns; power = 0.5 mJ; repetition rate = 1 Hz) with an OPO system. The absorption spectra and decay dynamics were obtained with a transmission configuration at λ_ab_ = 6000–1000 cm^–1^, and a diffuse reflection configuration at λ_ab_ = 15 000–6000 cm^–1^. The time resolution of this spectrometer was limited to 1–2 μs by the amplifier bandwidth (SR560, 1 MHz, Stanford Research Systems).

### ESR Measurement

Electron spin resonance measurements of C_3_N_4_ powders before and after plasma treatment were performed using the EMXplus instrument (Bruker) at the Institute for Molecular Science. The sample powders were placed in a quartz tube for measurements. The measurements were performed at room temperature using a microwave in the X-band range (9.6 GHz) at 5.024 mW. The center of the magnetic field was set to 3520 G, with a sweep width of 200 G. The receiver gain, modulation amplitude, and attenuation were set to 30 dB, 1.5 G, and 16 dB, respectively. The scan number and modulation frequency were set to 10 and 100 kHz, respectively. A Hg–Xe lamp (SLS400, Thorlabs) was used to obtain ESR spectra under light irradiation with a cold filter (CLDF-50S, Sigmakoki) for UV–vis light irradiation (750 nm > λ_ex_ > 360 nm) and a red dichroic filter (DIF-50S-RED, Sigmakoki) for red light irradiation (λ_ex_ > 550 nm). The output powers of the lamp through the filters were 64 mW and 69 mW for the UV–vis and red light, respectively.

### Cyclic Voltammetry Measurement

Cyclic voltammograms of **Ru(cat)** were obtained from an Ar-saturated DMA solution containing **Ru(cat)** (0.5 mM) and Et_4_NBF_4_ (0.1 M) as the supporting electrolyte using an electrochemical analyzer (ALS CHI-620Ex). A glassy carbon (diameter: 3 mm) working electrode, Ag/AgNO_3_ (10 mM) reference electrode, and a Pt counter electrode were used. The scan rate was 100 mV/s.

### Motto–Schottky Analysis

C_3_N_4_ powder was deposited on fluorine-doped tin oxide (FTO)-coated glass by drop casting a C_3_N_4_ dispersion in ethylene glycol. The electrode was heated in air at 473 K for 6 h. Impedance measurements were then performed using an electrochemical analyzer (BAS CHI760) in DMA. The C_3_N_4_-deposited FTO glass electrode was used as the working electrode with a Ag/AgNO_3_ reference electrode (10 mM), Pt wire counter electrode, and 0.1 M Et_4_NBF_4_ (99%, Sigma-Aldrich) supporting electrolyte. Mott–Schottky plots were acquired at frequencies of 500 and 1000 Hz.

## Results and Discussion

### Plasma Surface Modification of C_3_N_4_

**Figure 1:**
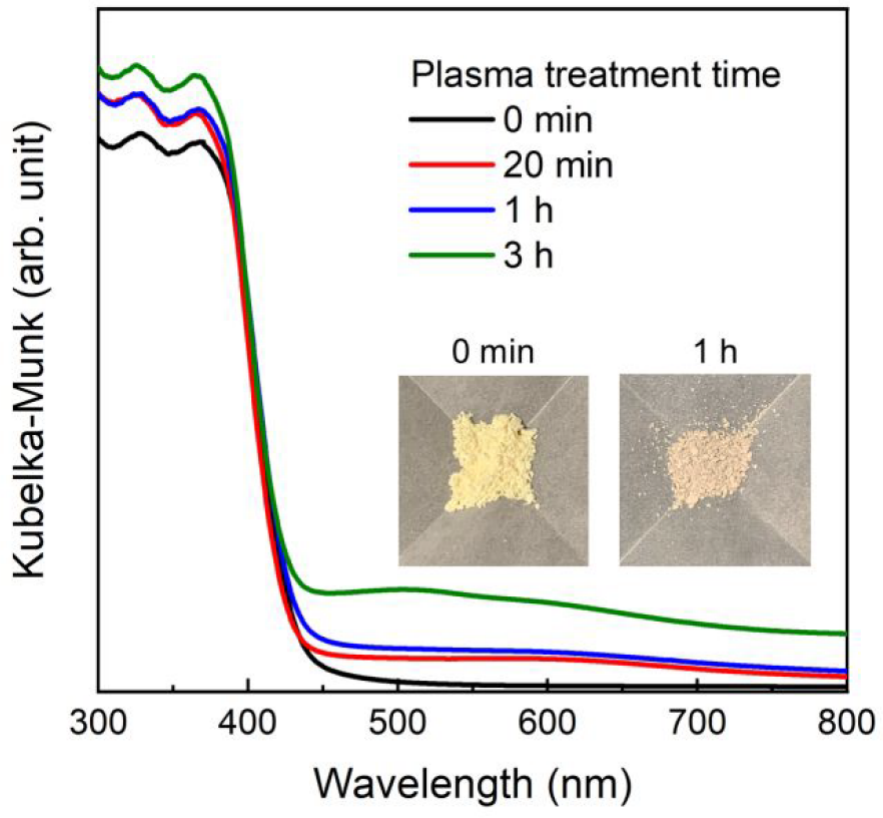
Diffuse reflectance absorption spectra of C_3_N_4_ before and after the different durations of plasma treatment. The inset photographs exhibit the color of C_3_N_4_ before and after the 1 h plasma treatment.

The C_3_N_4_ nanosheets were synthesized by heating urea at 823 K for 2 h in air.^29^ The plasma surface modification of C_3_N_4_ was performed under previously reported plasma conditions:^52^ nonequilibrium plasma was generated in 100 mL of 0.1 g L^−1^ NaCl aqueous solution containing 4 wt % hydroquinone, by applying 1.2 kV of bipolar pulsed voltage at 80 kHz to tungsten rod electrodes (1 mm in diameter) placed with 2 mm gap. The current–voltage (*I*–*V*) characteristics and a photograph of the plasma are shown in Figure S1. As observed from optical emissions of atomic oxygen and hydrogen (Figure S1b), the plasma provided a highly reactive environment. Hydroquinone was used as a precursor to introduce both oxygen functional groups and a carbon layer onto the C_3_N_4_ surface. 0.2 g of C_3_N_4_ powders was dispersed in an aqueous solution and the plasma was maintained. Figure 1 shows the diffuse reflectance absorption spectra of C_3_N_4_ before and after plasma treatment. A small increase in absorption in the visible region was observed after plasma treatment for 20 min and 1 h compared to that before plasma treatment, and this absorption conspicuously increased after 3 h of plasma treatment. In this study, we chose a plasma treatment time of 1 h to avoid disrupting the visible light absorption by the photosensitizer unit of **RuRu′**. Hereinafter, the C_3_N_4_ samples after 1 h of plasma treatment are referred to as plasma-C_3_N_4_.

**Figure 2:**
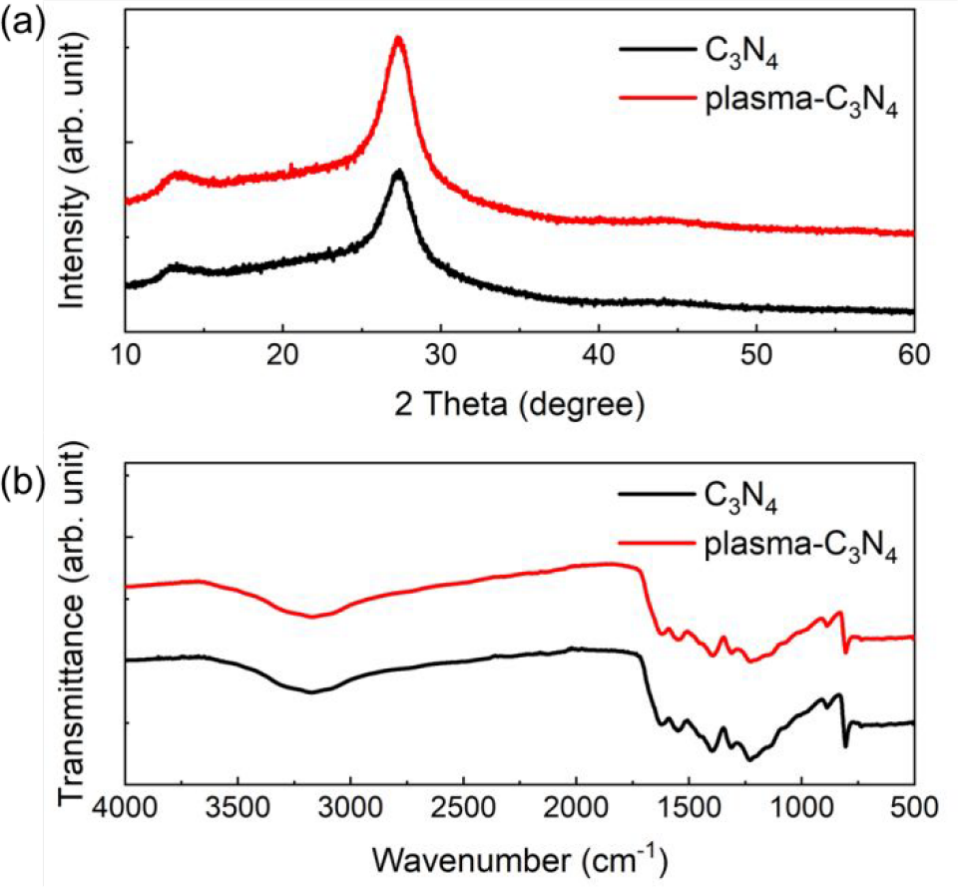
(a) XRD and (b) FT-IR spectra of C_3_N_4_ before and after the plasma treatment.

To analyze the effect of the plasma treatment of C_3_N_4_, X-ray diffraction (XRD) patterns, Fourier transform infrared (FT-IR) spectra, and thermogravimetric (TG) signals were obtained before and after the plasma treatment (Figure 2a, b and Figure S2). XRD patterns (Figure 2a) showed a peak at 13.1° that originated from the in-plane tri-s-triazine repeating units that form the backbone structure of C_3_N_4_, and another peak at 27.2° from the interlayer stacking of the conjugated backbone structure.^60^ In the FT-IR spectra (Figure 2b), the peaks at 808 cm^–1^ and 1200–1700 cm^–1^ are attributed to an out-of-plane bending mode of tri-s-triazine units and stretching modes in C_3_N_4_, respectively.^46^ TG signals (Figure S2) demonstrated thermal decomposition of C_3_N_4_ at 500–600 °C. Notably, in the XRD, FT-IR, and TG characterizations, there were no noticeable differences between the results observed before and after the plasma treatment (Figure 2a, b, and Figure S2). This strongly indicates that the crystal and chemical structures and thermal stability of C_3_N_4_ were maintained after the plasma treatment. The wavelength of the absorption edge in the diffuse reflectance absorption spectra was also maintained (Figure 1), demonstrating that the bandgap energy of C_3_N_4_ did not change. Therefore, the bulk properties of C_3_N_4_, such as its crystal structure, bandgap energy, chemical bonds, and thermal stability, were not strongly affected by the 1 h plasma treatment.

**Figure 3:**
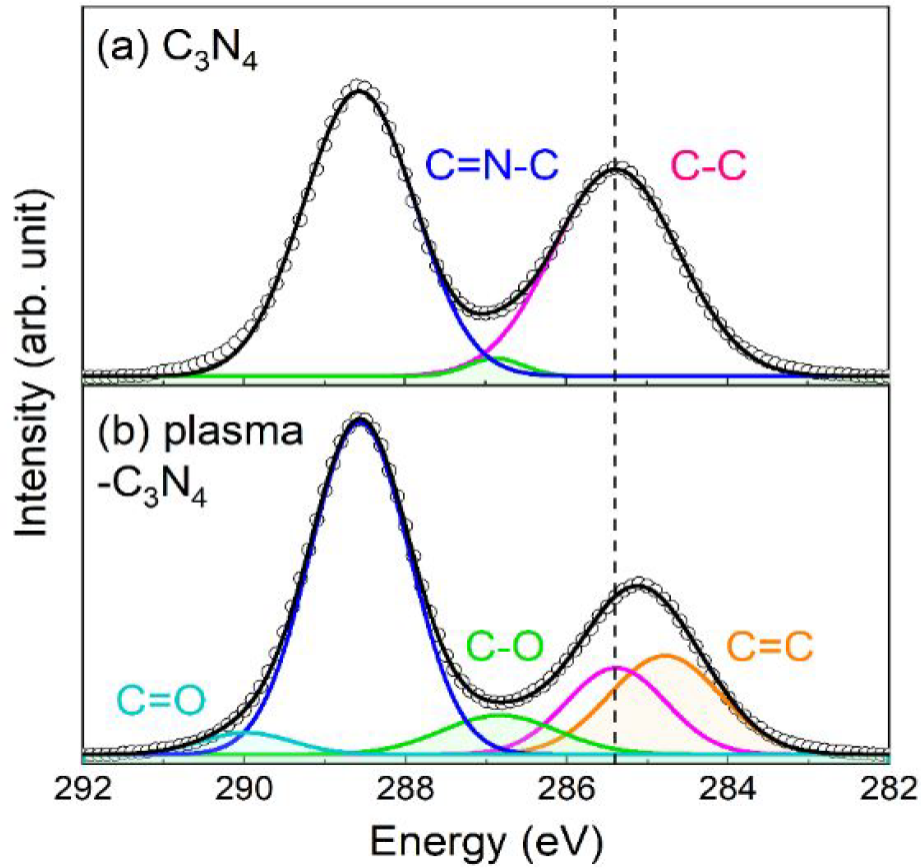
C 1s XPS spectra of C_3_N_4_ (a) before and (b) after the plasma treatment. The circular black dots indicate experimental data, while the Gaussian curves correspond to sp^3^ C–C (pink), C–O (light green), C=N–C (blue), sp^2^ C=C (orange), and C=O (sky blue). The resultant fitting curve (black) is depicted by solid lines.

The color of C_3_N_4_ changed from light yellow to light purple, and a small increase in the absorption in the visible region was observed (Figure 1). The Brunauer–Emmett–Teller (BET) surface area (*S*_BET_) estimated from the N_2_ adsorption–desorption isotherms was decreased by the plasma treatment, i.e., *S*_BET_ = 45 m^2^ g^–1^ and 39 m^2^ g^–1^ before and after the 1 h plasma treatment, respectively (Figure S3). These results suggest that the surface of C_3_N_4_ was modified by the plasma treatment. The C 1s XPS spectra of C_3_N_4_ before and after the plasma treatment showed different spectral shapes (Figure 3). The spectrum before the plasma treatment (Figure 3a) can be fitted by two dominant components at 288.6 and 285.4 eV attributed to the tri-s-triazine (N–C=N) structure and contaminant graphitic carbon (C–C), which is usually used for energy calibration, respectively.^61^ On the other hand, C=O (290.0 eV), C–O (286.9 eV), and C=C (284.8 eV) are additionally necessary to fit the spectrum after the plasma treatment (Figure 3b).^62^ There are two differences between the C 1s spectra before and after the plasma treatment: the first is an increase in the absorption of oxygen-containing groups (C–O, C=O), and second is the appearance of sp^2^ double-bonded carbon absorption (C=C). The area ratio of the oxygen-containing groups increased from 1.7% to 11% after plasma treatment. Therefore, carbon layers with abundant oxygen-containing functional groups were likely deposited on the surface of C_3_N_4_ through plasma treatment. It is noteworthy that the similar plasma treatment of multiwalled carbon nanotubes and boron nitrides also introduced oxygen-containing groups onto the surfaces of these materials^52,53^ The shapes of the N 1s XPS spectra were the same before and after plasma treatment (Figure S4), indicating that the chemical states of nitrogen atoms on C_3_N_4_ were not strongly influenced by plasma treatment.

**Figure 4:**
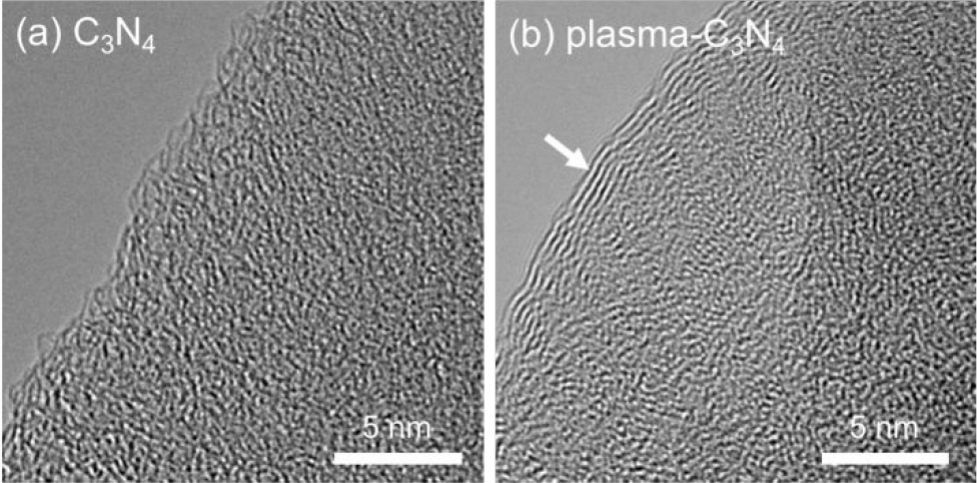
TEM images of C_3_N_4_ (a) before and (b) after plasma treatment. White arrows indicate the deposited carbon layer around the edge of C_3_N_4_.

The deposition of the carbon layers was also observed by TEM measurements (Figure 4): stripe patterns appeared uniformly around the edge of plasma-C_3_N_4_ only after the plasma treatment, while the disordered sp^2^ morphology of C_3_N_4_ was maintained before and after the plasma treatment.^63,64^ Therefore, the broad absorption in the visible region (Figure 1) is attributable to the deposition of graphene-like carbon layers with extended π conjugation. The deposition of carbon layers might contribute to the decrease in *S*_BET_ owing to the increase in the thickness of the nanosheet structure of C_3_N_4_. Based on the observations described above, the mechanism of the plasma surface modification can be described as follows: hydroquinone was adsorbed onto the C_3_N_4_ powders by π–π stacking, and subsequently, the plasma caused oxidative polymerization of the hydroquinone molecules to form oxygen-rich carbon stacking layers.

### Affinity of Metal Complexes with Methyl Phosphonic Acid Anchoring Groups for C_3_N_4_

**Figure 5:**
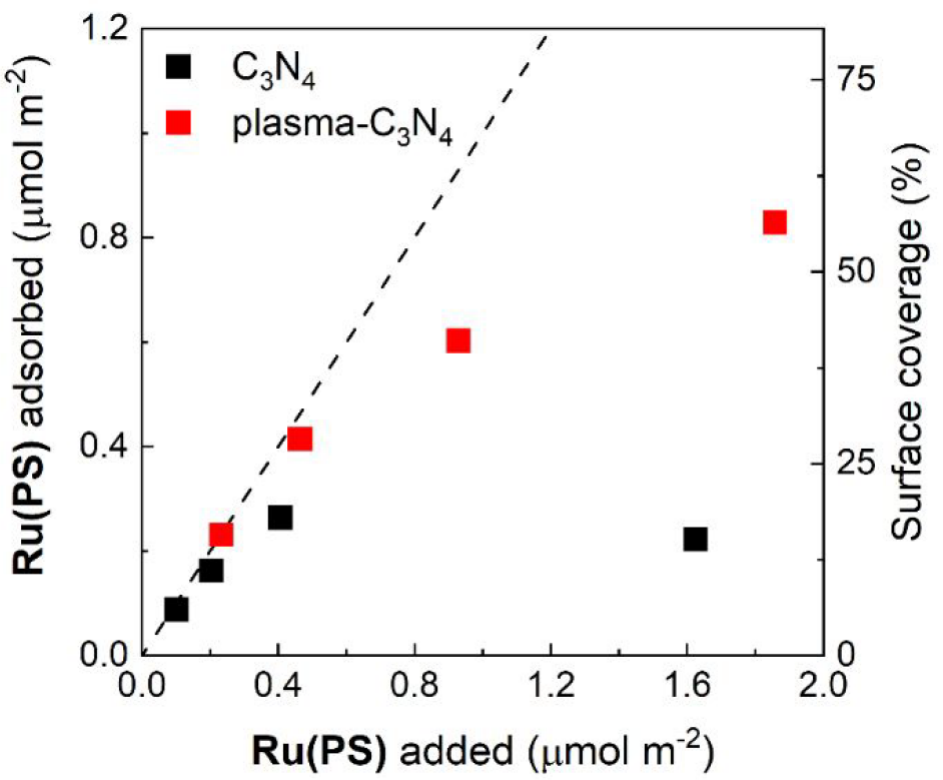
Adsorption density and surface coverage of **Ru(PS)** on C_3_N_4_ before and after the plasma treatment. The dashed line indicates the total amount of added **Ru(PS)** in the solution for the adsorption.

Figure 5 and Figure S5 show the amount of **Ru(PS)** (Chart 1) adsorbed on C_3_N_4_ before and after plasma treatment, where **Ru(PS)** was used as a model complex of the photosensitizer unit of **RuRu′** to investigate the adsorption characteristics of molecules with methyl phosphonic acid anchoring groups on C_3_N_4_. For adsorption, C_3_N_4_ was dispersed in an acetonitrile (MeCN) solution containing the PF_6_ salts of **Ru(PS)** under dim light for 5 days. Then, the filtered solid was washed several times with MeCN. The plasma-C_3_N_4_ showed 3-fold better adsorption capacity (≤0.83 μmol m^–2^) than nontreated C_3_N_4_ (≤0.25 μmol m^–2^): the maximum adsorption corresponded to 56% and 18% of surface coverage for plasma-C_3_N_4_ and nontreated C_3_N_4_, respectively. The improved adsorption was further confirmed by the diffuse reflectance spectra of the C_3_N_4_ powders after the adsorption of **Ru(PS)** (Figure S6), where the absorption peak at λ_max_ = 460 nm is due to the singlet metal-to-ligand charge transfer (^1^MLCT) absorption of **Ru(PS)**. The adsorption improvement is attributed to the abundant formation of oxygen functional groups on plasma-C_3_N_4_, which acted as adsorption sites for the methyl phosphonic acid groups of **Ru(PS)**.^42^ The superior adsorption capacity of plasma-C_3_N_4_ also proved to be true in the case of adsorption of **RuRu′** instead of **Ru(PS)**. The maximum adsorption capacity of 0.86 μmol m^–2^ corresponded to a surface coverage of 58%.

### Photocatalytic CO_2_ Reduction by **RuRu′**/Ag/Plasma-C_3_N_4_ and RuRu′/Ag/C_3_N_4_

To prepare the hybrid photocatalyst, 1.25 wt % of Ag was first loaded onto plasma-C_3_N_4_ via impregnation and subsequent H_2_ reduction at 473 K—it has been reported that Ag loading accelerates the photocatalysis of C_3_N_4_ owing to its role as an electron sink.^28,65^ Ag-loaded plasma-C_3_N_4_ and nontreated C_3_N_4_ were dispersed in MeCN solutions containing **RuRu′** under dim light for 5 days to adsorb 0.5 μmol g^–1^ of **RuRu′**, which is similar to the method for making **Ru(PS)**-adsorbed C_3_N_4_. The adsorbed amount of **RuRu′** was also estimated with inductively coupled plasma-optical emission spectroscopy (ICP-OES). This indicated that 0.74 μmol of Ru (ions) was attached on 1 g of **RuRu′**/Ag/C_3_N_4_, i.e., 0.37 μmol g^–1^ of **RuRu′** adsorption, which reasonably agreed with the value described above. For testing the desorption of the adsorbed **RuRu′** from both C_3_N_4_ samples, the nontreated and plasma-C_3_N_4_, both of which were adsorbed with both 0.5 μmol g^–1^ of **RuRu′** and 1.25 wt % of Ag particles, (**RuRu′**/Ag/C_3_N_4_ and **RuRu′**/Ag/plasma-C_3_N_4_) were added to *N,N’*-dimethylacetamide (DMA)/triethanolamine (TEOA) (4:1, v:v) and stirred for 90 h under dim light. The desorption ratios were 3.0% and 2.5% for nontreated C_3_N_4_ and plasma-C_3_N_4_, respectively. Therefore, the strong adsorption of **RuRu′** on C_3_N_4_ was maintained after plasma surface modification.

The prepared hybrid photocatalyst, **RuRu′**/Ag/plasma-C_3_N_4_, was dispersed in DMA/TEOA (4:1, v:v) solutions via stirring under a CO_2_ atmosphere and then irradiated at λ_ex_^max^ = 410 and 460 nm (LED lights), which are suitable wavelengths for the band gap excitation of C_3_N_4_ and excitation of the photosensitizer ruthenium unit of **RuRu′**, respectively (Figure S7).

**Figure 6:**
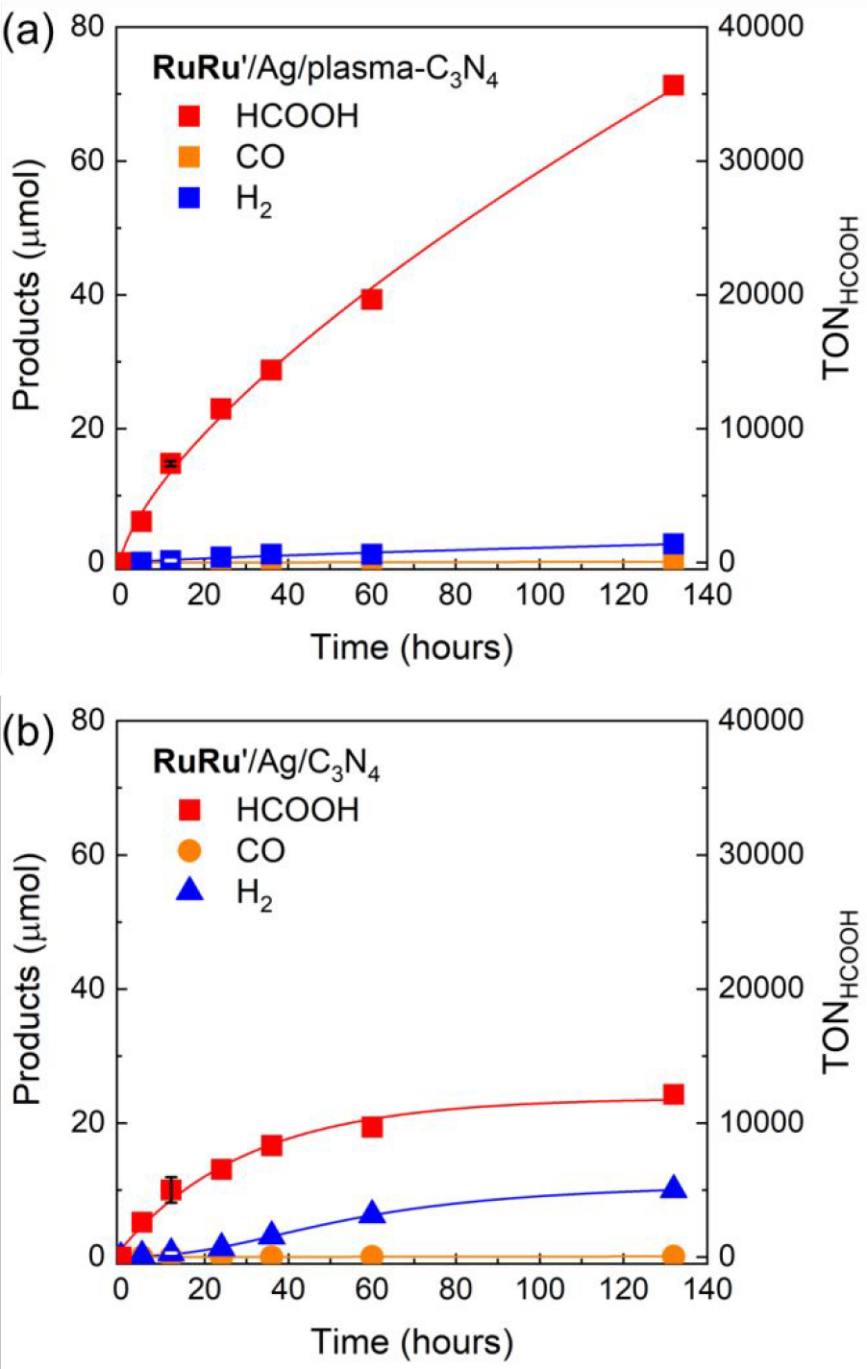
Time courses of the products during the photocatalytic reactions using (a) **RuRu′** (0.5 μmol g^–1^)/Ag (1.25 wt %)/plasma-C_3_N_4_ and (b) **RuRu′** (0.5 μmol g^–1^)/Ag (1.25 wt %)/C_3_N_4_. Photocatalytic reactions were performed in DMA/TEOA (4:1, v/v) solutions with stirring under a CO_2_ atmosphere by visible light irradiation using LEDs at λ_ex_^max^ = 410 and 460 nm. Experimental error bars of formation of HCOOH and H_2_ after 12 h of irradiation are also shown as results of standard deviations for four experiments.

HCOOH was produced as the major product along with very small amounts of H_2_ and CO during irradiation. The turnover number for HCOOH production (TON_HCOOH_) based on **RuRu′** used exceeded 35000 after 132 h of light irradiation (Figure 6a). The selectivity of HCOOH production (Selectivity_HCOOH_) was 99% after 5 h of irradiation (Table 1, entry 1) and remained at over 96% even after 132 h of irradiation (Figure 6a). The apparent quantum yield (AQY) for photocatalytic HCOOH production with **RuRu′**/Ag/plasma-C_3_N_4_ was 1.6% when was measured using 400 nm monochromatic light from a 300 W xenon lamp.

**Table 1:** Photocatalytic CO_2_ Reduction and Control Experimental Results^a^

			products (μmol)				
entry	photocatalyst	absence	HCOOH	CO	H_2_	TON_HCOOH_	Selectivity_HCOOH_ /%
1	**RuRu′**/Ag/plasma-C_3_N_4_	—	6.4	N.D.	0.08	3171	99
2	**RuRu′**/Ag/C_3_N_4_	—	5.4	N.D.	0.20	2680	96
3	**RuRu′**/Ag/plasma-C_3_N_4_	CO_2_^b^	N.D.	N.D.	0.5	0	0
4	**RuRu′**/Ag/plasma-C_3_N_4_	TEOA	N.D.	N.D.	trace	0	0
5	**RuRu′**/Ag/plasma-C_3_N_4_	light irradiation	N.D.	N.D.	N.D.	0	0
6	Ag/plasma-C_3_N_4_	**RuRu′**	trace	N.D.	0.41	—	—
7	**RuRu′**/plasma-C_3_N_4_	Ag	0.37	0.01	0.01	185	94
8	**RuRu′**/C_3_N_4_	Ag	0.15	0.03	0.02	75	75
9	**Ru(cat)**/Ag/plasma-C_3_N_4_	photosensitizer unit	2.9	0.02	0.23	1471	92

a Reaction conditions: photocatalyst, 4 mg (Ru complex loading, 0.5 μmol g^–1^; Ag loading, 1.25 wt %); solution,: 4.0 mL of DMA/TEOA (4:1, v:v) with bubbled CO_2_; light source, 410 and 460 nm-centered LEDs; reaction time, 5 h.

b Under an Ar atmosphere.

In contrast, when using a hybrid photocatalyst with nontreated C_3_N_4_ (**RuRu′**/Ag/C_3_N_4_) instead of **RuRu′**/Ag/plasma-C_3_N_4_, the TON_HCOOH_ and Selectivity_HCOOH_ after 132 h of irradiation were much lower at 12 000 and 70%, respectively (Figure 6b). The rate of HCOOH production was also lower than that using **RuRu′**/Ag/plasma-C_3_N_4_ (Table 1, Figure 6). Therefore, the plasma surface modification of C_3_N_4_ drastically improved the photocatalytic activity of the hybrid photocatalyst for CO_2_ reduction. In addition, when **RuRu′**/Ag/C_3_N_4_ was used, the production of HCOOH decreased after 36 h of irradiation, accompanied by an increase in H_2_ production as a side product (Figure 6b, Table 1). The simultaneous increase in H_2_ production and suppression of HCOOH production suggest deactivation of **RuRu′**, resulting in H_2_ production from C_3_N_4_: C_3_N_4_ produces H_2_ under visible light irradiation through photocatalysis in the presence of TEOA as an electron donor.^12^ As shown in the XRD patterns (Figure S8), X-ray absorption fine structure (XAFS) spectra (Figure S9), and Ag 3d XPS spectra (Figure S10), C_3_N_4_ and Ag maintained their chemical structures even after the deactivation of **RuRu′**/Ag/C_3_N_4_, implying that C_3_N_4_ and Ag were not involved in the deactivation. On the other hand, after 132 h of light irradiation of **RuRu′**/Ag/plasma-C_3_N_4_, **RuRu′** was detached from Ag/plasma-C_3_N_4_ by stirring the photocatalytic powders in 10 mM NaOH aqueous solution and the UV–vis absorption spectrum of the filtrate was measured. The ^1^MLCT absorption of the photosensitizer unit of **RuRu′** was clearly red-shifted (Figure S11), which is a typical spectral change when ligand substitution of [Ru(diimine)_3_]^2+^-type photosensitizers proceeds to give the solvent complex, [Ru(diimine)_2_(solvent)]^2+^, that cannot work as a photosensitizer.^6,22^ Therefore, the decomposition of the photosensitizer unit of **RuRu′** should be one cause of the deactivation of the hybrid photocatalysts.

Experimental errors of formation of HCOOH and H_2_ after 12 h of irradiation were evaluated by standard deviation for four experiments, i.e., 17% and 2.7% for H_2_ and HCOOH production with **RuRu′**/Ag/plasma-C_3_N_4_ (Figure 6a), and 6.9% and 19% for H_2_ and HCOOH production with **RuRu′**/Ag/C_3_N_4_ (Figure 6b). The error evaluation clearly verified the superior photocatalytic activity of **RuRu′**/Ag/plasma-C_3_N_4_.

Table 1 summarizes the photocatalytic reactions of **RuRu′**/Ag/plasma-C_3_N_4_ for 5 h under an Ar atmosphere (entry 3), without TEOA (entry 4), without light irradiation (entry 5) and without **RuRu′** (entry 6). CO_2_, TEOA, light irradiation, and **RuRu′** were all necessary for photocatalytic CO_2_ reduction in the hybrid system. The Ag loading on C_3_N_4_ was effective for improvement of the HCOOH production activity of the hybrid even after the plasma surface modification of C_3_N_4_ (Table 1, entries 1 and 7), as previously demonstrated that Ag loading can promote photocatalytic CO_2_ reduction activity by sintering photoexcited electrons onto the surface of C_3_N_4_.^28,65^ Plasma surface modification also improved the photocatalytic CO_2_ reduction activity in the hybrid photocatalyst even without Ag loading (Table 1, entries 7 and 8), with a 2.5-fold increase in turnover number of HCOOH formation and an improved selectivity from 75% to 94%. Therefore, the improvement of the photocatalytic CO_2_ reduction activity of the hybrid photocatalyst with plasma-C_3_N_4_ is essentially a positive effect of plasma surface modification with or without Ag loading. The advantage of the Z-scheme photoexcitation of **RuRu′**/Ag/plasma-C_3_N_4_ was investigated by comparing with a system using only the **Ru(cat)** mononuclear catalyst (Chart 1), which is a model complex of the catalytic unit of **RuRu′**,^29^ instead of **RuRu′**. The turnover number of HCOOH formation in the case of **Ru(cat)**/Ag/plasma-C_3_N_4_ (TON_HCOOH_ = 1471, entry 9) was less than half of that in the case of **RuRu′**/Ag/plasma-C _3_N_4_ (TON_HCOOH_ = 3171, entry 1).

**Figure 7:**
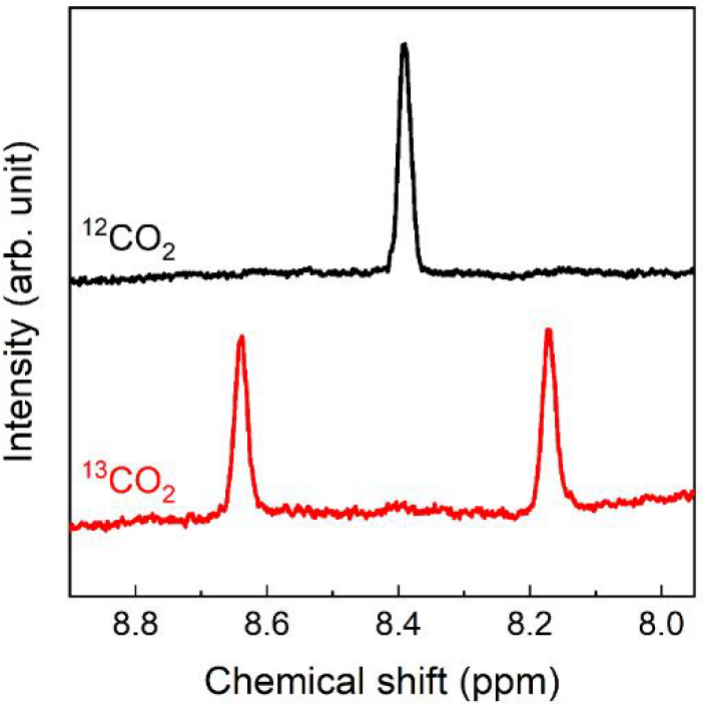
^1^H NMR spectra of DMA/TEOA solutions after the photocatalytic reactions using **RuRu′** (0.5 μmol g^–1^)/Ag (1.25 wt %)/plasma-C_3_N_4_. The photocatalyst powders were removed by filtration before the measurement. The suspension of **RuRu′**/Ag/plasma-C_3_N_4_ was irradiated at λ_ex_^max^ = 410 and 460 nm (LED light sources) for 40 h under 640 mmHg of ^13^CO_2_ (black) and saturated unlabeled CO_2_ (red).

To identify the carbon source of the produced HCOOH, isotope tracer techniques were performed using ^13^CO_2_. The filtrate solution after the photocatalytic reaction with **RuRu′**/Ag/plasma-C_3_N_4_ was analyzed using ^1^H NMR spectroscopy with the no-deuterium proton (No-D) technique (Figure 7). A doublet attributed to a mixture of H^13^COOH and H^13^COO^–^ was observed at δ = 8.4 ppm with *J* = 186 Hz (red),^26^ while only a very small singlet signal attributable to a mixture of H^12^COOH and H^12^COO^–^ was detected when the photocatalytic reaction was performed under ^13^CO_2_ atmosphere. On the other hand, in the case of the photocatalytic reaction under an unlabeled CO_2_ atmosphere, only a singlet was observed at 8.4 ppm (black). Therefore, we can conclude that the carbon source for the produced HCOOH was CO_2_.

**Figure 8:**
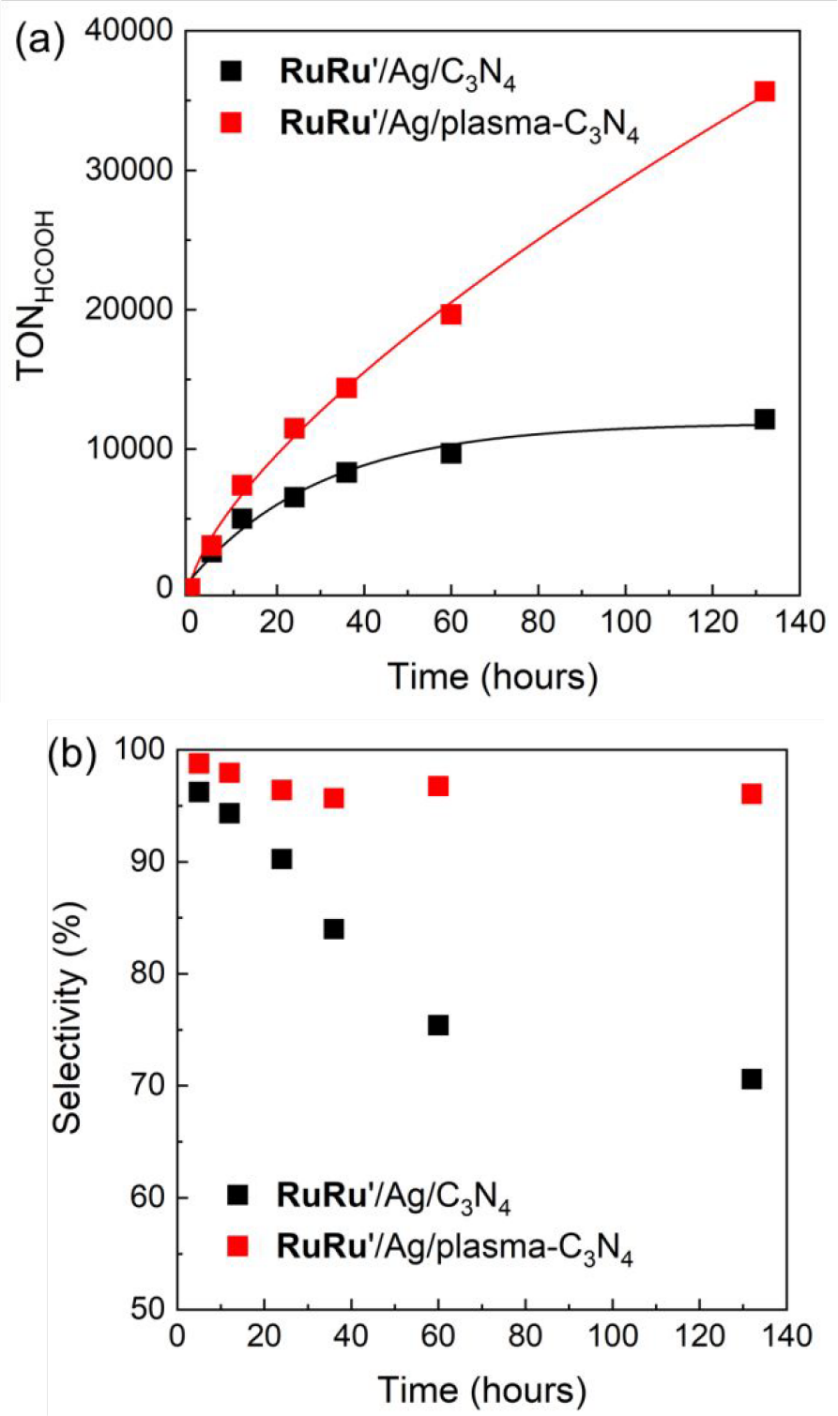
(a) Turnover number for HCOOH production and (b) product selectivity during the photocatalytic CO_2_ reduction with **RuRu′** (0.5 μmol g^–1^)/Ag (1.25 wt %)/plasma-C_3_N_4_ and **RuRu′** (0.5 μmol g^–1^)/Ag (1.25 wt %)/C_3_N_4_. The photocatalytic reactions were performed in DMA/TEOA (4:1, v/v) with stirring under CO_2_ atmosphere by visible light irradiation using LEDs at λ_ex_^max^ = 410 and 460 nm.

As summary in the photocatalytic reaction part, a comparison of the photocatalytic CO_2_ reduction of **RuRu′**/Ag/plasma-C_3_N_4_ and **RuRu′**/Ag/C_3_N_4_ clarifies two prominent advantages of the plasma surface modification of C_3_N_4_. First, as shown in Figure 8a, the plasma surface modification of C_3_N_4_ greatly boosted the durability of photocatalytic CO_2_ reduction by three times, which is attributed to the suppression of **RuRu′** deactivation. Second, the product selectivity of the photocatalytic CO_2_ reduction was significantly improved (Figure 8b); a very high selectivity for HCOOH production (over 96%) was achieved even after 132 h of irradiation using **RuRu′**/Ag/plasma-C_3_N_4_, while the selectivity severely decreased to 71% in the system using **RuRu′**/Ag/C_3_N_4_.

The amount of **RuRu′** was optimized to investigate the potential photocatalytic durability of **RuRu′**/Ag/plasma-C_3_N_4_. When the adsorption amount of **RuRu′** on **RuRu′**/Ag/plasma-C_3_N_4_ was decreased to 0.13 μmol g^–1^, a TON_HCOOH_ of 50000 was recorded after 132 h of irradiation (Figure S12). This is the highest turnover number achieved by a metal-complex/semiconductor hybrid system so far.^28^ In this system, a certain amount of H_2_ was produced as a byproduct probably because an excess amount of electrons accumulated in the plasma-C_3_N_4_ and/or Ag owing to the low density of adsorbed **RuRu′**.

### Effects of Plasma Surface Modification on Photophysical and Photochemical Properties of C_3_N_4_

**Figure 9:**
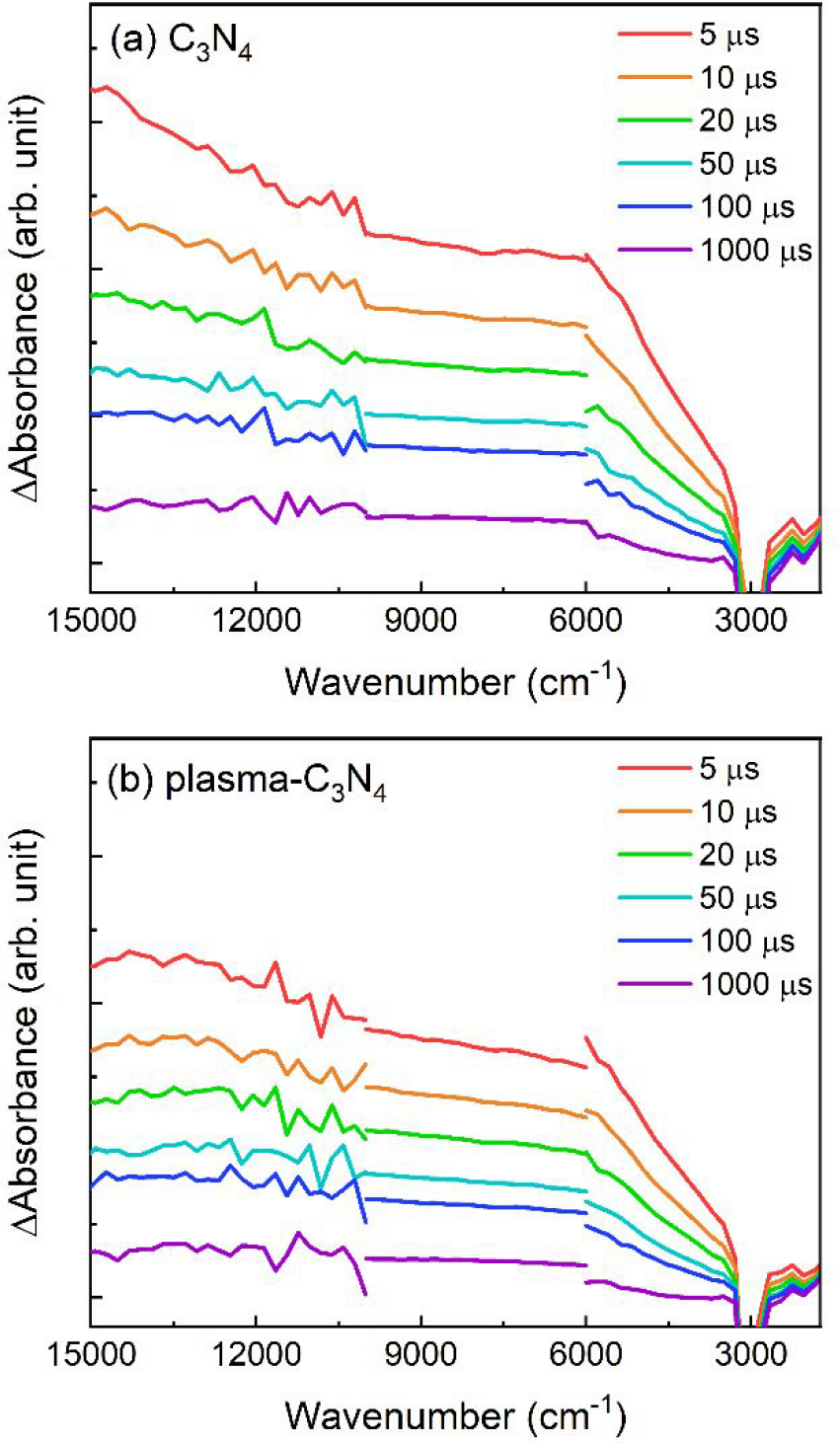
Time-resolved infrared absorption spectra of C_3_N_4_ (a) before and (b) after plasma treatment.

Time-resolved infrared absorption spectroscopy (TR-IR)^28,59^ was employed to investigate the effects of the plasma surface modification of C_3_N_4_ on the photophysical properties, and in particular to clarify the dynamics of the photoexcited carriers in the C_3_N_4_ powder; upon the bandgap photoexcitation of C_3_N_4_ using 420 nm laser pulse for a 6 ns duration, absorption of photogenerated charged carriers occurred, followed by infrared light absorption by the sample. Powders of C_3_N_4_ or plasma-C_3_N_4_ deposited on a CaF_2_ plate were placed in a vacuum cell purged with N_2_ gas. The absorption spectra and decay dynamics were obtained using a transmission configuration at λ_ab_ = 6000–1000 cm^–1^ and a diffuse reflection configuration at λ_ab_ = 15 000–6000 cm^–1^. Broad absorption was observed from 15 000 to 1000 cm^–1^ (Figure 9).

**Figure 10:**
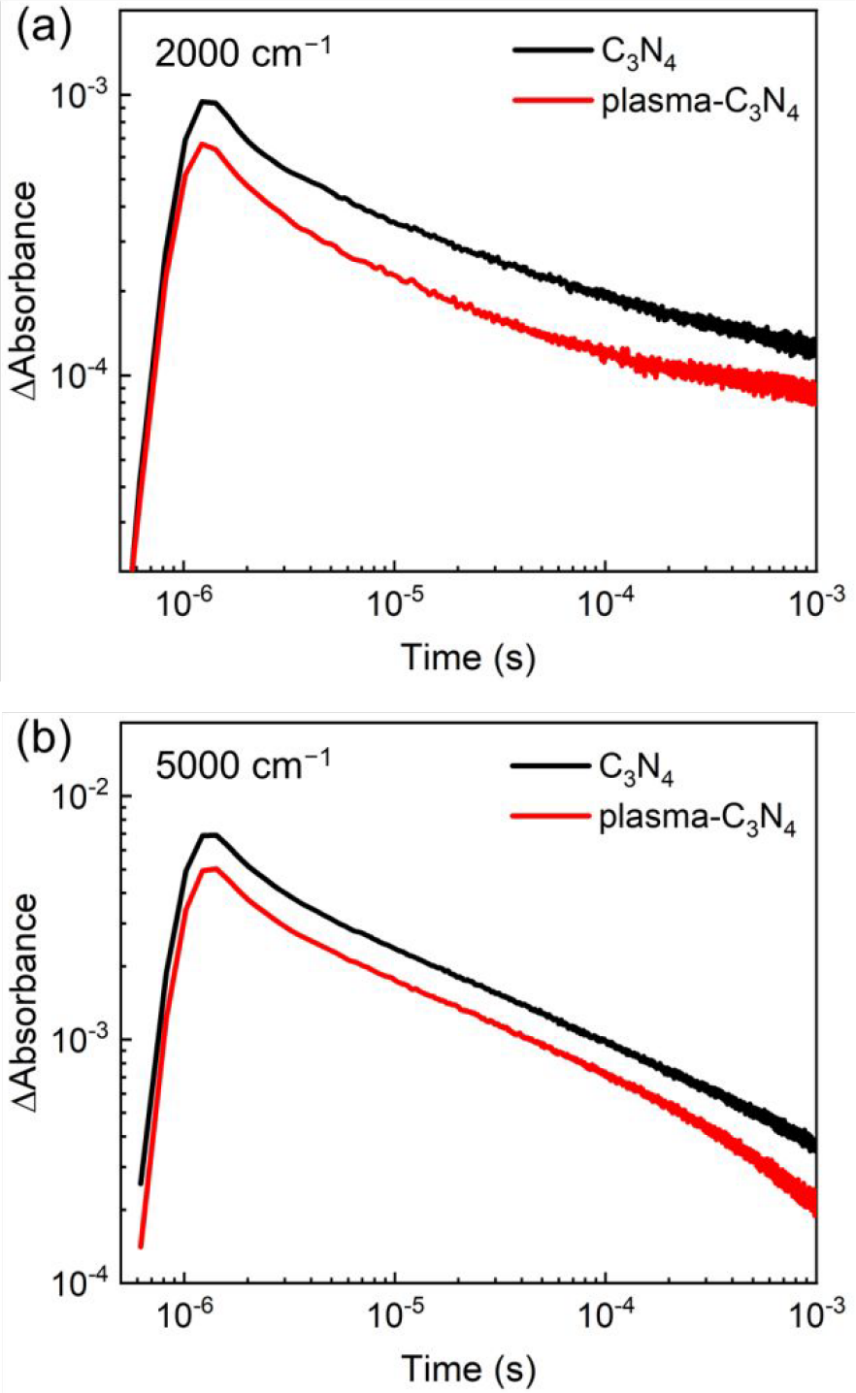
Decay curves of infrared absorbance of C_3_N_4_ before and after the plasma treatment, monitored at (a) 2000 cm^–1^ and (b) 5000 cm^–1^.

Absorption bands appearing at 15000–3000 cm^–1^ are attributable to electrons trapped in the deep defect sites of C_3_N_4_, while absorption at 3000–1000 cm^–1^ is assigned to free electrons and/or electrons trapped in the shallow defect sites of C_3_N_4._^28,59,66,67^ In both regions, the shapes of the absorption spectra of C_3_N_4_ and plasma-C_3_N_4_ were very similar, indicating that the types of photoexcited carriers and their levels of entrapment in C_3_N_4_ were not drastically changed by the plasma surface modification. The decay kinetics of free and shallowly trapped electrons and electrons trapped in deep defect sites were investigated by recording the changes in the transient absorption intensity at 2000 and 5000 cm^–1^, respectively (Figure 10). The decay curves were fitted using three exponential functions; the lifetimes are listed in Table 2. The lifetimes τ_1_ and τ_2_ of plasma-C_3_N_4_ were shorter by ∼30–50% at 2000 cm^–1^ compared to those of C_3_N_4_, while all of the lifetimes measured at 5000 cm^–1^ were similar. Therefore, the plasma surface modification slightly shortens the lifetime of only the free or shallowly trapped electrons produced by band gap photoexcitation.

**Table 2:** Lifetimes (τ_n_) and Pre-exponential Factors (*A*_*n*_) of the Infrared Absorbance Decays of C_3_N_4_ and Plasma-C_3_N_4_ Obtained by Fitting with Three Exponential Functions

wavenumber (cm^–1^)	sample	τ_1_ (μs)	*A*_1_	τ_2_ (μs)	*A*_2_	τ_3_ (ms)	*A*_3_
2000	C_3_N_4_	2.7	0.72	63	0.14	3.9	0.14
	plasma-C_3_N_4_	1.9	0.76	37	0.13	4.0	0.11
5000	C_3_N_4_	3.3	0.74	70	0.17	1.7	0.09
	plasma-C_3_N_4_	3.8	0.73	87	0.18	1.4	0.09

**Figure 11:**
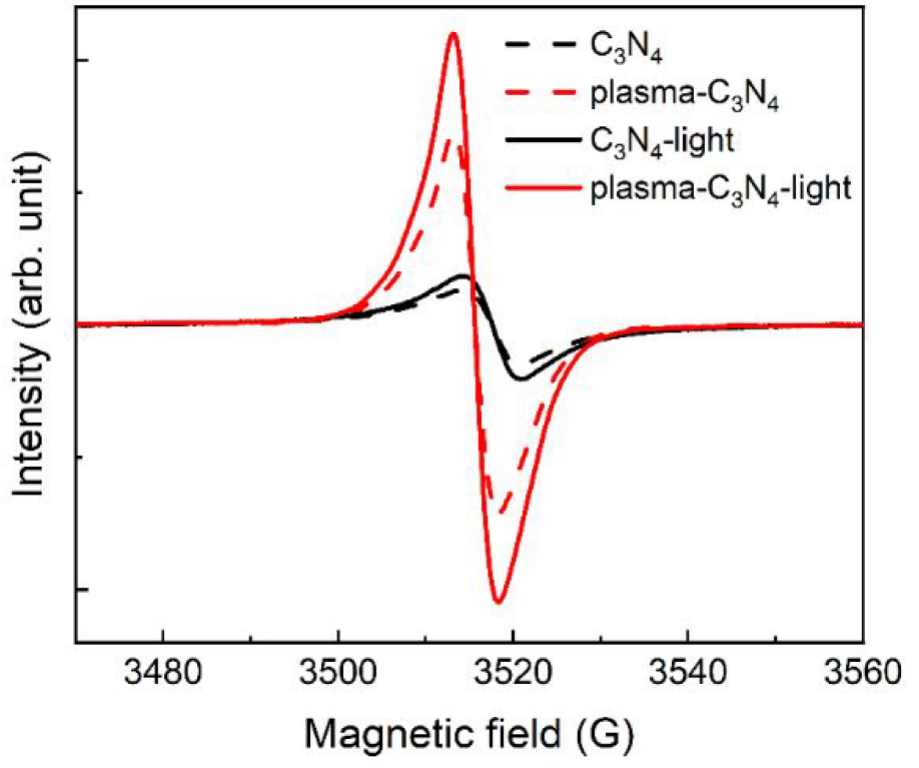
ESR spectra of C_3_N_4_ before and after the plasma treatment measured in the dark (dashed lines) or under UV–vis light irradiation at 750 nm > λ_ex_ > 360 nm (solid lines).

Electron spin resonance (ESR) measurements were performed in the X-band region to investigate the defect sites on C_3_N_4_ and plasma-C_3_N_4_. A single Lorentzian peak was observed at *g* = 2.0043 for C_3_N_4_ and at *g* = 2.0047 for plasma-C_3_N_4_, as shown in Figure 11. The *g* values of the peaks correspond to unpaired electrons of carbon that are localized in the π conjugation of C_3_N_4_ and/or amorphous carbons.^68^ An increase in the signal intensity of plasma-C_3_N_4_ indicates that the plasma treatment significantly increased the surface defects. UV–vis light irradiation (750 nm > λ_ex_ > 360 nm) increased the signal intensities in both C_3_N_4_ and plasma-C_3_N_4_ (Figure 11), while the signal intensities did not change under red light (λ_ex_ > 550 nm) irradiation (Figure S13a); the increase in intensity under UV–vis light irradiation was more pronounced for plasma-C_3_N_4_. This suggests that photoexcited electrons accumulated more efficiently in the surface defect sites on plasma-C_3_N_4_ than those on C_3_N_4_ under steady light irradiation. This is consistent with the results of the TR-IR measurement as the surface defects on plasma-C_3_N_4_ should cause shorter lifetimes for free and/or shallowly trapped electrons. Note that the *g*-factor is a parameter that depends on the environment in which the electron spin exists. The higher *g* value of plasma-C_3_N_4_ compared to C_3_N_4_ suggests that the surface defects formed by the plasma surface modification differed from the original defects on C_3_N_4_, and the environment of the electron spin changed owing to the deposition of the carbon layer on the C_3_N_4_ produced by plasma surface modification. It has been reported that surface oxygen functional groups on graphitic carbon materials such as carbon dots and reduced graphene oxide fixed on titanium oxide can trap and accumulate photoexcited electrons because oxygen functional groups confine electrons by decreasing the electronic delocalization of sp^2^ hybridization.^69,70^ Therefore, the oxygen functional groups produced on plasma-C_3_N_4_ should have caused the surface defects. In addition to the oxygen functional groups, heterostructure of graphitic-C_3_N_4_ and carbon layer might also contribute to the accumulation of photoexcited electrons on plasma-C_3_N_4_.^71^ Further investigation such as time-resolved terahertz spectroscopy^72^ is necessary to clarify this point. ESR measurements of Ag-loaded plasma-C_3_N_4_ (Ag/plasma-C_3_N_4_) also demonstrated an increase in signal intensity, a higher *g* value, and a more pronounced increase in signal intensity under UV–vis light irradiation compared to the ESR signals of Ag/C_3_N_4_ (Figure S13b) indicating that the accumulation of photoexcited electrons in surface defect sites on plasma-C_3_N_4_ occurs even with Ag loading.

**Figure 12:**
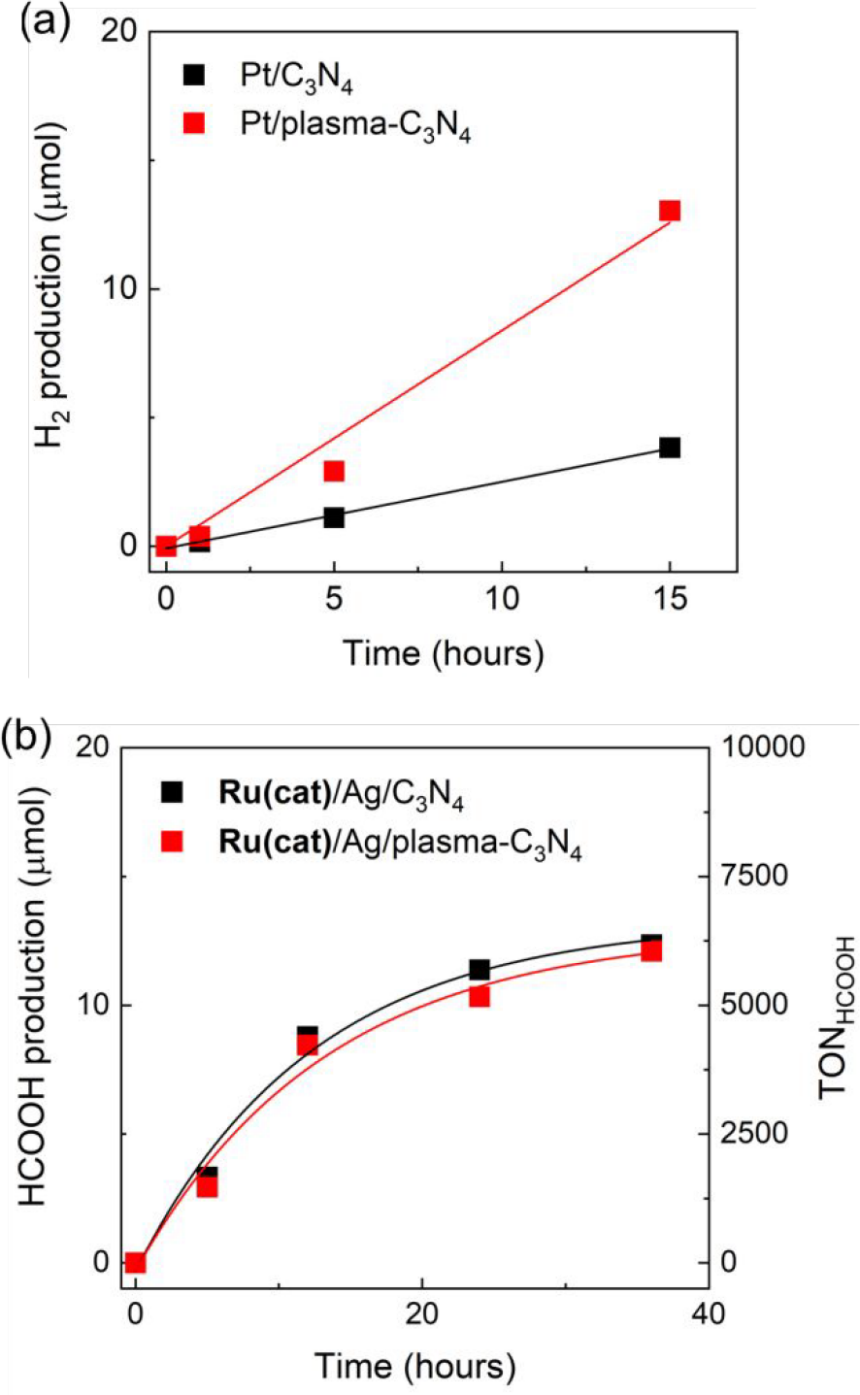
Time courses of (a) H_2_ in photocatalytic H_2_ production with Pt(3 wt %)/plasma-C_3_N_4_ or Pt(3 wt %)/C_3_N_4_, and (b) HCOOH in photocatalytic CO_2_ reduction with **Ru(cat)** (0.5 μmol g^–1^)/Ag(1.25 wt %)/plasma-C_3_N_4_ or **Ru(cat)** (0.5 μmol g^–1^)/Ag(1.25 wt %)/C_3_N_4_. The photocatalytic reactions were performed in H_2_O/TEOA (4:1, v/v) (a) under an Ar or (b) CO_2_ atmosphere by irradiation using LEDs at λ_ex_^max^ = 410 and 460 nm.

The accumulation of photoexcited electrons in the surface defect sites in plasma-C_3_N_4_ was also supported by the results of photocatalytic H_2_ production experiments performed in an Ar-saturated H_2_O/TEOA (4:1, v:v) solution using C_3_N_4_ or plasma-C_3_N_4_ on which 3 wt % platinum (Pt) cocatalyst was deposited (Pt/C_3_N_4_, Pt/plasma-C_3_N_4_). Pt-loaded C_3_N_4_ is known to efficiently photocatalyze H_2_ evolution;^12^ photoexcited electrons in the conduction band move to the loaded Pt and subsequently produce H_2_, while holes in the valence band are consumed by the oxidation of TEOA. Figure 12a shows that Pt/plasma-C_3_N_4_ produced H_2_ more efficiently (more than twice) than Pt/C_3_N_4_. This strongly supports the increase in the number of photoexcited electrons on plasma-C_3_N_4_ owing to its surface defect sites.

We also attempted to use Ag/plasma-C_3_N_4_ with 0.5 μmol g^–1^ of **Ru(cat)** as a catalyst (**Ru(cat)**/Ag/plasma-C_3_N_4_) for photocatalytic CO_2_ reduction, which acts only through the excitation of plasma-C_3_N_4_, i.e., not via the Z-scheme.^57^ In contrast to the H_2_ production described above, the photocatalytic CO_2_ reduction activity of **Ru(cat)**/Ag/plasma-C_3_N_4_ was slightly lower than that of **Ru(cat)**/Ag/C_3_N_4_, as shown in Figure 12b and Figure S14. The reduction potential of **Ru(cat)** was *E*_p_ = −1.56 V vs Ag/AgNO_3_ (Figure S15), while the conduction band minimum of C_3_N_4_ and plasma-C_3_N_4_ were approximately estimated to be −1.89 V and −1.84 V (vs Ag/AgNO_3_) respectively, as shown in the Motto–Schottky plots of C_3_N_4_ and plasma-C_3_N_4_ (Figure S16). Based on a comparison of these potentials, we can conclude that only free and shallowly trapped electrons on C_3_N_4_ can thermodynamically move to **Ru(cat)** and contribute to the CO_2_ reduction reaction by **Ru(cat)**. Therefore, the plasma surface modification of C_3_N_4_ slightly lowered the number of higher-energy free electrons and shallow-trapped electrons that can be directly transferred to **Ru(cat)**, while also drastically increasing the number of trapped electrons in deep surface defect sites, which can be used for H_2_ evolution and Z-scheme type electron transfer but cannot directly transfer to **Ru(cat)**.

**Scheme 2:**
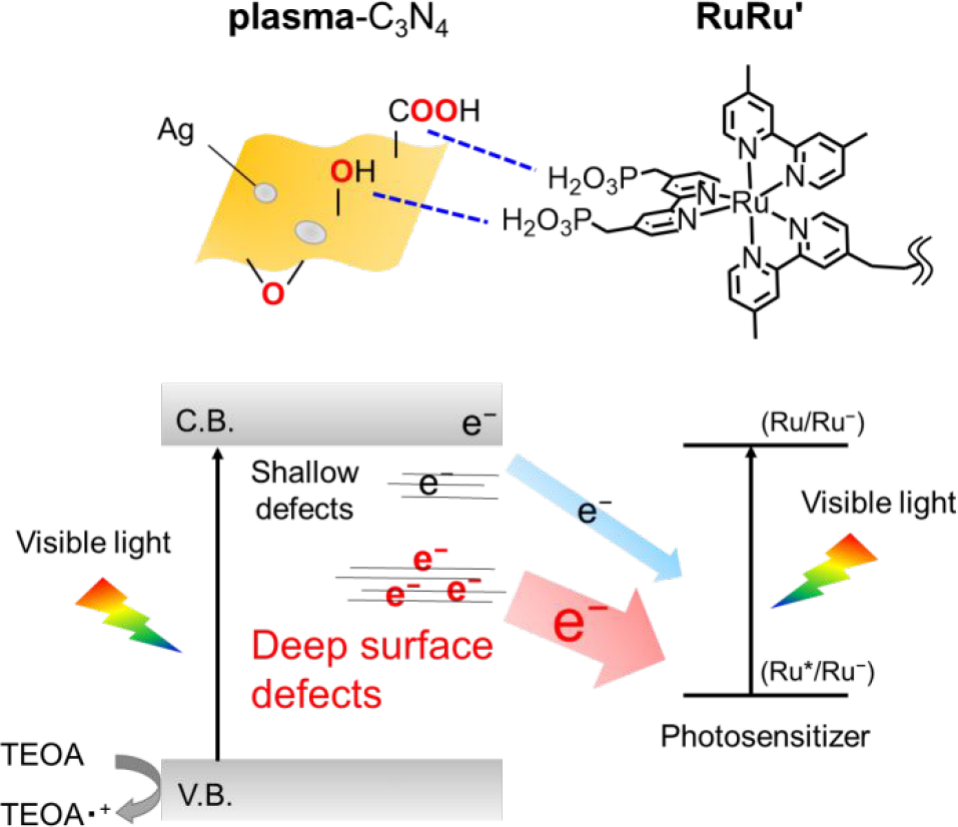
Interfacial electron flow in **RuRu′**/Ag/plasma-C_3_N_4_ during the photocatalytic reaction

Based on the investigation of the effect of the plasma surface modification of C_3_N_4_ on the dynamics, kinetics, and reactivities of the photoexcited electron, we can conclude that the oxygen-rich carbon stacking layers formed on C_3_N_4_ by the plasma surface modification work not only as adsorption sites for **RuRu′** but also as deep trapping sites to accumulate photoexcited electrons, which result in increased photocatalysis of the hybrid for CO_2_ reduction (Scheme 2). The increase in the electron supply to **RuRu′** should shorten the duration of the intermediate(s) made from the reduced **RuRu′** and CO_2_, prevent undesirable deactivation processes of **RuRu′**, and enhance the photocatalytic durability of **RuRu′**/Ag/plasma-C_3_N_4_. Prevention of the decomposition of **RuRu′** can also suppress H_2_ production from C_3_N_4_ by consuming photogenerated electrons for CO_2_ reduction, leading to very high HCOOH production selectivity over a long irradiation time.

The investigation of the positive effects of plasma surface modification clearly elucidated one of the prominent advantages of supramolecular photocatalysts in hybrid photocatalytic systems with semiconductor materials. Photoexcitation of the photosensitizer unit of **RuRu′** enabled the use of deeply trapped electrons on plasma-C_3_N_4_ due to the strong oxidation power of the photoexcited photosensitizer unit of **RuRu′** (Ru*/Ru^–^ = +0.17 V vs Ag/AgNO_3_),^22^ whereas the mononuclear metal complex catalyst (**Ru(cat)**) cannot receive such deeply trapped electrons owing to its much more negative reduction potential (−1.56 V vs Ag/AgNO_3_). Therefore, the effective usage of electron trap sites on semiconductors by supramolecular photocatalysts via Z-scheme photoexcitation should provide a new strategy for improving the interfacial electron transfer in hybrid photocatalysts.

## Conclusion

Plasma treatment in aqueous solutions containing hydroquinone “surface-selectively” modifies C_3_N_4_—an oxygen-rich carbon layer forms on C_3_N_4_ without affecting its bulk structure and properties. The plasma surface modification has proven to be a useful method for improving the interfacial affinity of metal complexes: the methyl phosphonic acid anchoring groups on C_3_N_4_ increased the amount of **RuRu′** adsorbed on C_3_N_4_ by three times compared to that of nontreated C_3_N_4_. Plasma treatment also enhanced the photocatalytic ability of the hybrid photocatalyst (**RuRu′**/Ag/plasma-C_3_N_4_), with a 3-fold enhancement in the turnover number of HCOOH production (>50 000) and a high product selectivity of HCOOH production compared to the nontreated hybrid (**RuRu′**/Ag/C_3_N_4_). The mechanism for these enhancements in photocatalysis is explained by the acceleration of electron transfer from plasma-C_3_N_4_ to the excited **RuRu′** owing to the high accumulation of photoexcited electrons in the surface defect sites in the oxygen-rich carbon layer of plasma-C_3_N_4_. These results also provide notable insights into the advantages of supramolecular photocatalysts owing to the deeply trapped electrons in semiconductors using the Z-scheme type electron transfer. The plasma surface modification can be used to modify not only C_3_N_4_ but also other organic semiconductors, to introduce active sites that can combine with various functional molecules without altering the bulk properties of the semiconductors and without losing (and maybe even improving) their photocatalytic activity.

## Supporting Information Available

*I*–*V* characteristics and optical emission spectrum of the plasma, diffuse reflectance absorption spectra, TG signals, N_2_ adsorption measurement, XPS spectra, adsorption isotherm of **Ru(PS)**, LED spectrum, XRD pattern, XANES spectra, Ag K-edge EXAFS spectra, FT of EXAFS spectra, time-resolved infrared absorption spectra, ESR spectra, photocatalytic CO_2_ reduction, and CV and Mott–Schottky plots (PDF)
