# Supplementary material for: Surface-Specific Modification of Graphitic Carbon Nitride by Plasma for Enhanced Durability and Selectivity of Photocatalytic CO2 Reduction with a Supramolecular Photocatalyst
Source: ACS Appl Mater Interfaces. 2023 Mar 1;15(10):13205–18. doi: 10.1021/acsami.3c00955 (PMC10020964; doi:10.1021/acsami.3c00955)
Supplement: Supplementary file 1 — am3c00955_si_001.pdf [file am3c00955_si_001.pdf]

Supporting Information

Surface-specific modification of graphitic carbon nitride by plasma for enhanced durability and selectivity of photocatalytic CO_2 reduction with a supramolecular photocatalyst

Noritaka Sakakibara,[1, 2] Mitsuhiko Shizuno,[1] Tomoki Kanazawa,[2, 3] Kosaku Kato,[4] Akira Yamakata,[4] Shunsuke Nozawa,[3] Tsuyohito Ito,[5] Kazuo Terashima,[5] Kazuhiko Maeda,[1*] Yusuke Tamaki,[1] Osamu Ishitani[1, 6*]

[1] Department of Chemistry, School of Science, Tokyo Institute of Technology, 2-12-1-NE-2 Ookayama, Meguro, Tokyo 152-8550, Japan

[2] Japan Society for the Promotion of Science, Kojimachi Business Center Building, 5-3-1 Kojimachi, Chiyoda, Tokyo 102-0083, Japan

[3] Institute of Materials Structure Science, High Energy Accelerator Research Organization, Tsukuba, Ibaraki 305-0801, Japan

[4] Faculty of Natural Science and Technology, Okayama University, 3-1-1, Tsushima-naka, Kita-ku, Okayama, 700-8530, Japan

[5] Department of Advanced Materials Science, Graduate School of Frontier Sciences, The University of Tokyo, 5-1-5 Kashiwanoha, Kashiwa, Chiba 277-8561, Japan

[*]E-mail: kazuo@plasma.k.u-tokyo.ac.jp, maedak@chem.titech.ac.jp, ishitani@chem.titech.ac.jp

[6] Department of Chemistry, Graduate School of Advanced Science and Engineering, Hiroshima University,

1-3-1 Kagamiyama, Higashi-Hiroshima, Hiroshima 739-8526, Japan

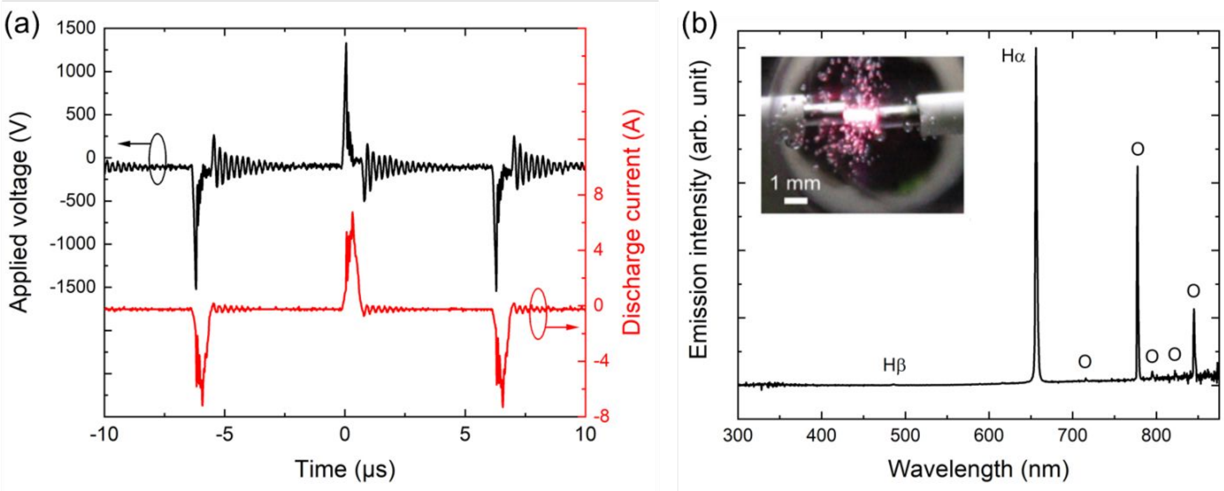

Figure S1 (a) *I*–*V* characteristics and (b) optical emission spectrum of the plasma. The inset image in (b) is a photograph of the plasma. The plasma was generated between tungsten electrodes by applying the pulsed voltage shown in Figure S1(a).

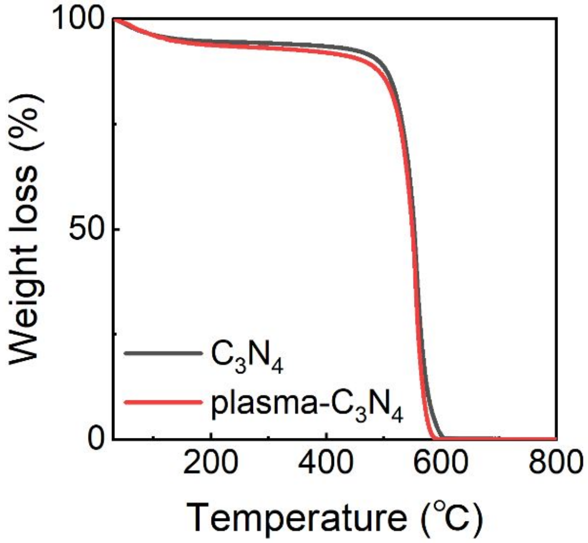

Figure S2 TG signals of C_3N_4 before and after plasma treatment. Measurements were conducted in air with a 5 °C min^{-1} ramp rate.

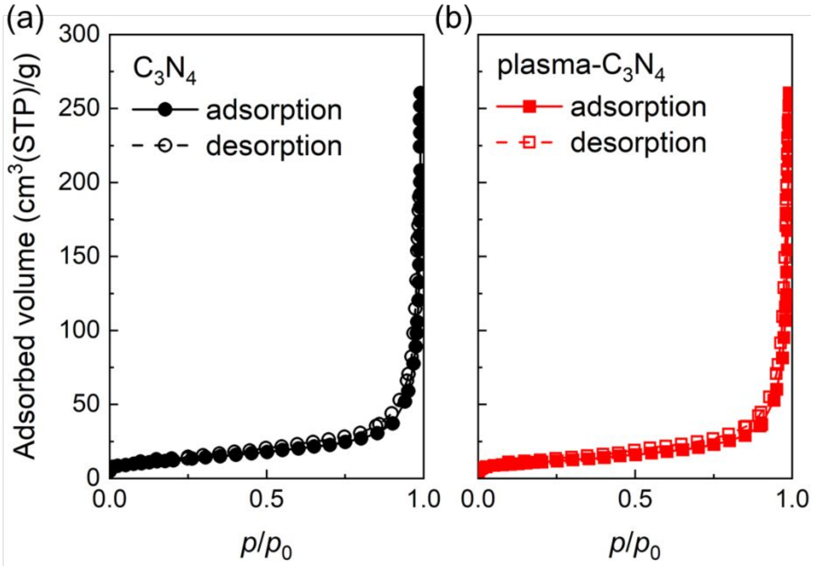

Figure S3 N_2 adsorption–desorption isotherms of C_3N_4 (a) before and (b) after plasma treatment.

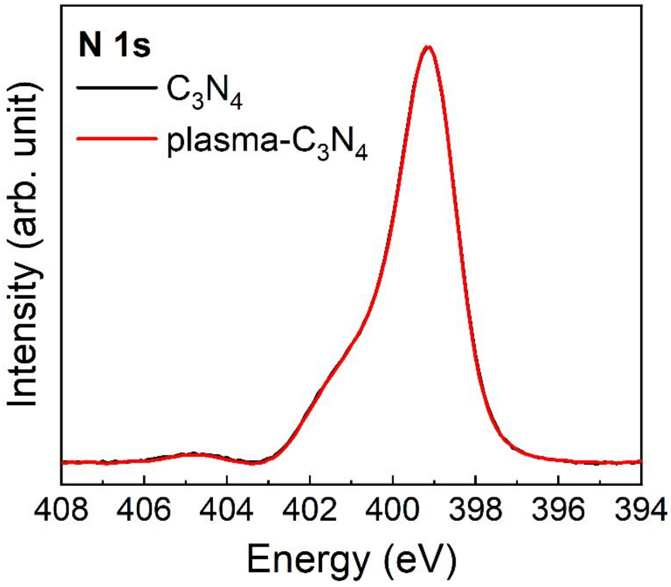

Figure S4 N 1s XPS spectra of C_3N_4 before and after plasma treatment.

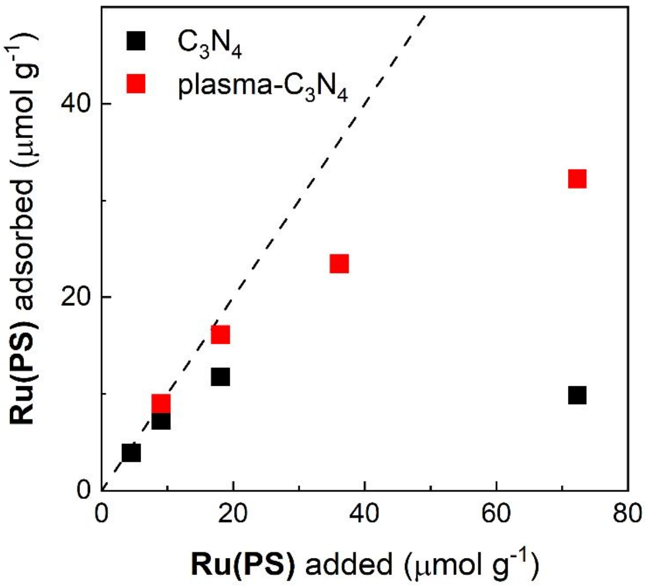

Figure S5 Adsorption amount of **Ru(PS)** on C_3N_4 before and after plasma treatment. The dashed line indicates the total amount of **Ru(PS)** added to the solution for the adsorption process. Note that the adsorption density and surface coverage in Figure 4 were calculated using the BET surface area assuming uniform adsorption. For the calculation of surface coverage, the short molecular radius of **Ru(PS)**, 0.6 nm, was utilized for the size of the **Ru(PS)** on C_3N_4, based on MM2 molecular mechanics program calculations.[34]

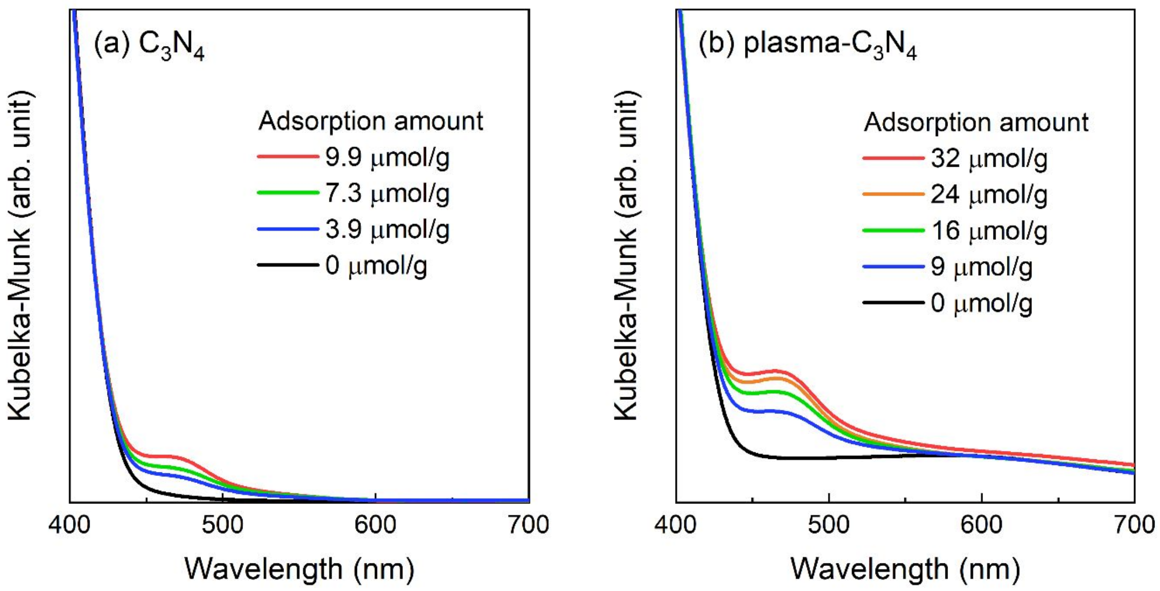

Figure S6 Diffuse reflectance absorption spectra of (a) **Ru(PS)**/C_3N_4 and (b) **Ru(PS)**/plasma-C_3N_4.

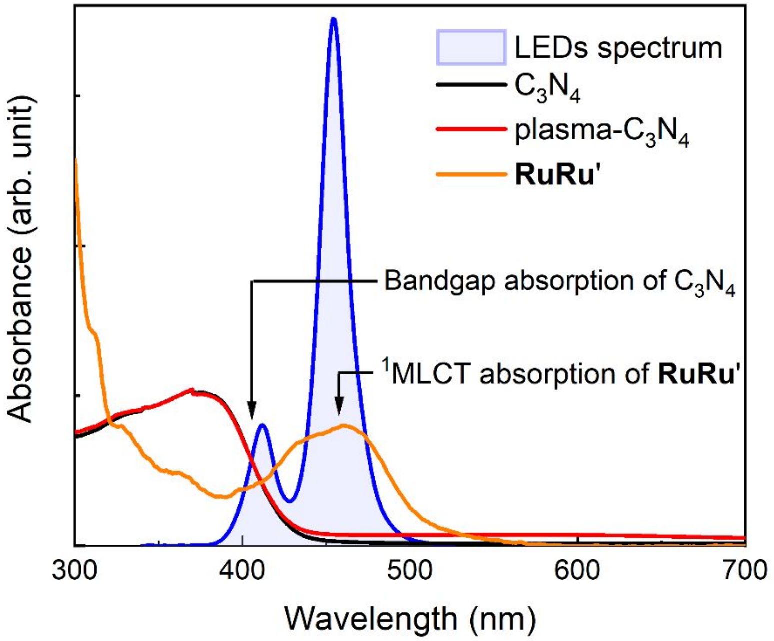

Figure S7 Spectrum of light irradiation used for photocatalytic CO_2 reduction, with 410 nm and 460 nm centered-LEDs. Kubelka–Munk conversed diffuse reflectance absorption spectra of C_3N_4 and plasma-C_3N_4 and absorption spectrum of **RuRu'** measured in MeCN are also displayed. The 410-nm centered-LED was used for photoexcitation of C_3N_4 by bandgap absorption, while the 460-nm centered-LED was used for photoexcitation of the photosensitizer unit of **RuRu'** by ^{1}MLCT absorption.

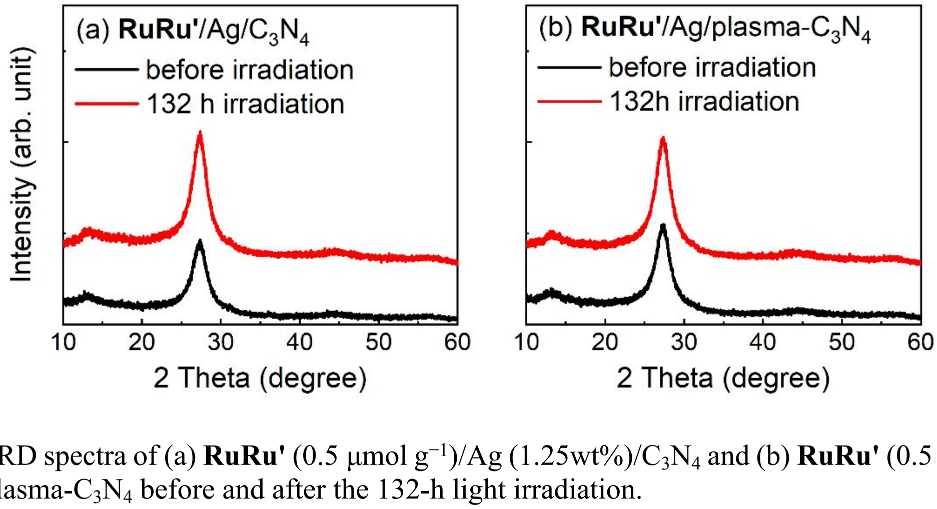

Figure S8 XRD spectra of (a) **RuRu'** (0.5 μmol g^{-1})/Ag (1.25wt%)/C_3N_4 and (b) **RuRu'** (0.5 μmol g^{-1})/Ag (1.25wt%)/plasma-C_3N_4 before and after the 132-h light irradiation.

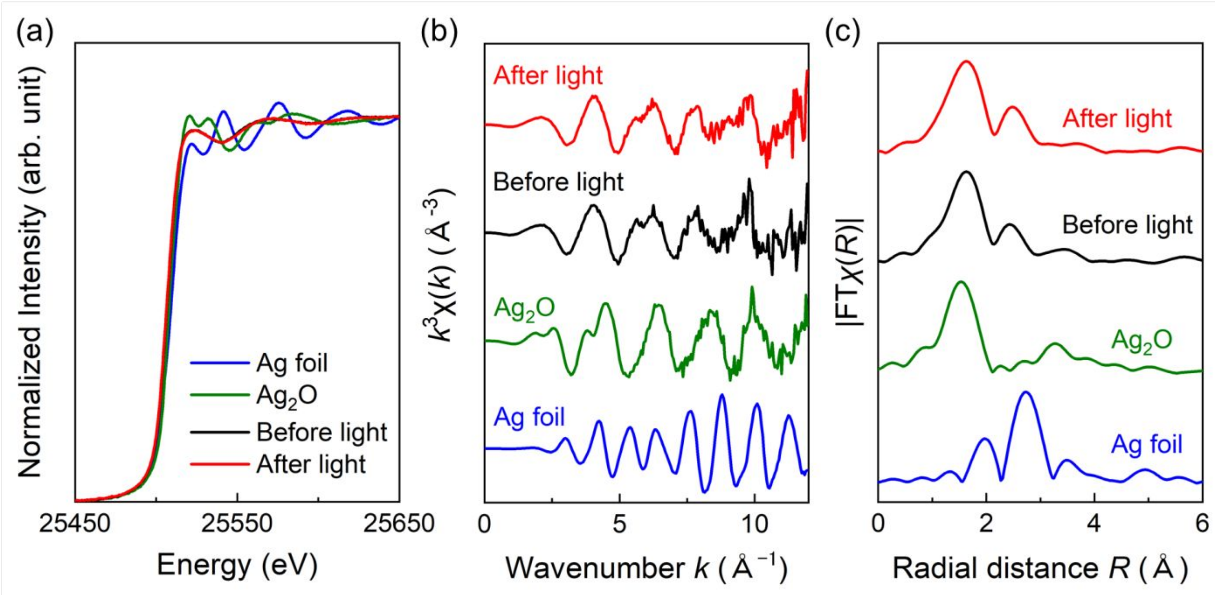

Figure S9 (a) XANES spectra, (b) Ag K-edge EXAFS spectra and (c) FT of EXAFS spectra of **RuRu'** (0.5 μmol g^{-1})/Ag (1.25wt%)/C_3N_4 before and after light irradiation. The spectra of Ag foil and Ag_2O are also shown as a reference. Ag in the hybrid photocatalyst can be assigned as Ag_2O with some contribution of Ag^0, although other Ag species should contribute to the structure because the absorption edge structure of **RuRu'**/Ag/C_3N_4 cannot be reproduced perfectly by the superposition of the reference spectra of Ag and Ag_2O.[65] Anyway, the structure of Ag in the hybrid photocatalyst was maintained after the photocatalytic reaction

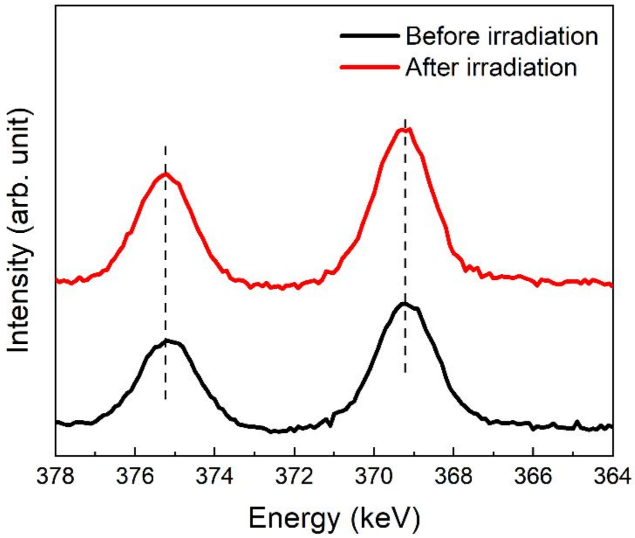

Figure S10 Ag 3d XPS spectra of **RuRu'**(0.5 μmol g^{-1})/Ag(1.25wt%)/C_3N_4 before and after the 132 h light irradiation.

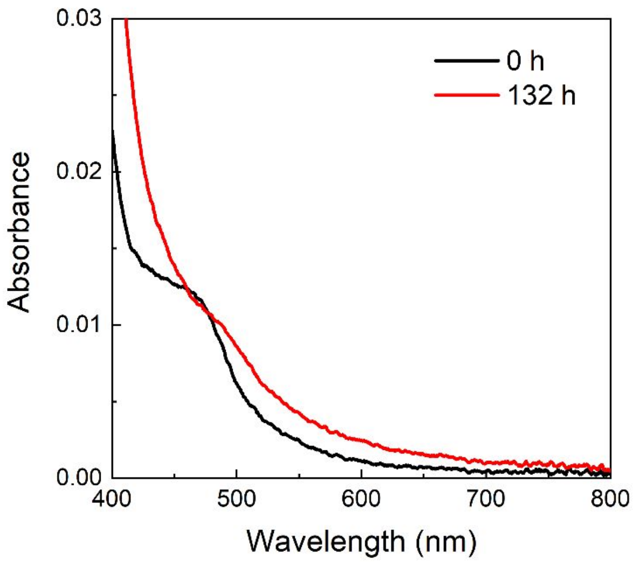

Figure S11 UV-vis absorption spectra of 10 mM NaOH aqueous solution containing **RuRu'** detached from **RuRu'**/Ag/plasma-C_3N_4 powders before and after the 132-h light photocatalytic reaction.

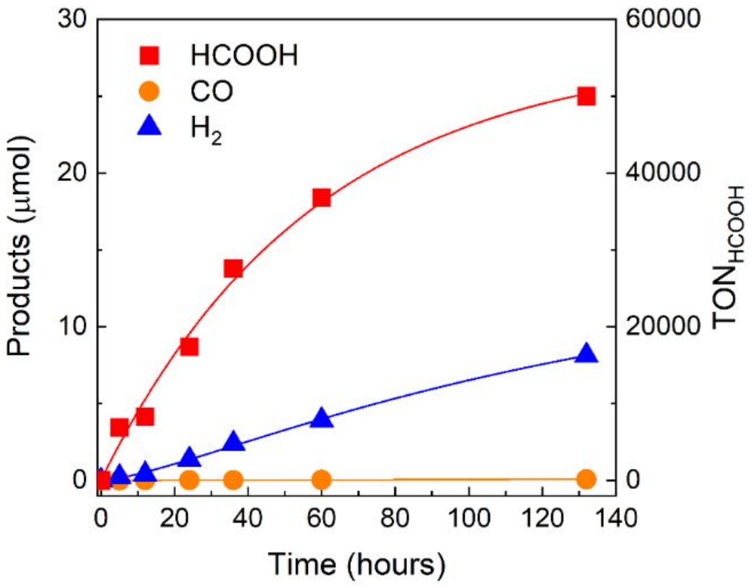

Figure S12 Turnover number of HCOOH production and product selectivity during the photocatalytic CO_2 reduction with **RuRu'** (0.13 μmol g^{-1})/Ag (1.25wt%)/plasma-C_3N_4. Photocatalytic reactions were performed in DMA/TEOA (4:1, v/v) solutions with stirring under CO_2 atmosphere by visible light irradiation using LEDs at λ_{ex}^{max} = 410 and 460 nm.

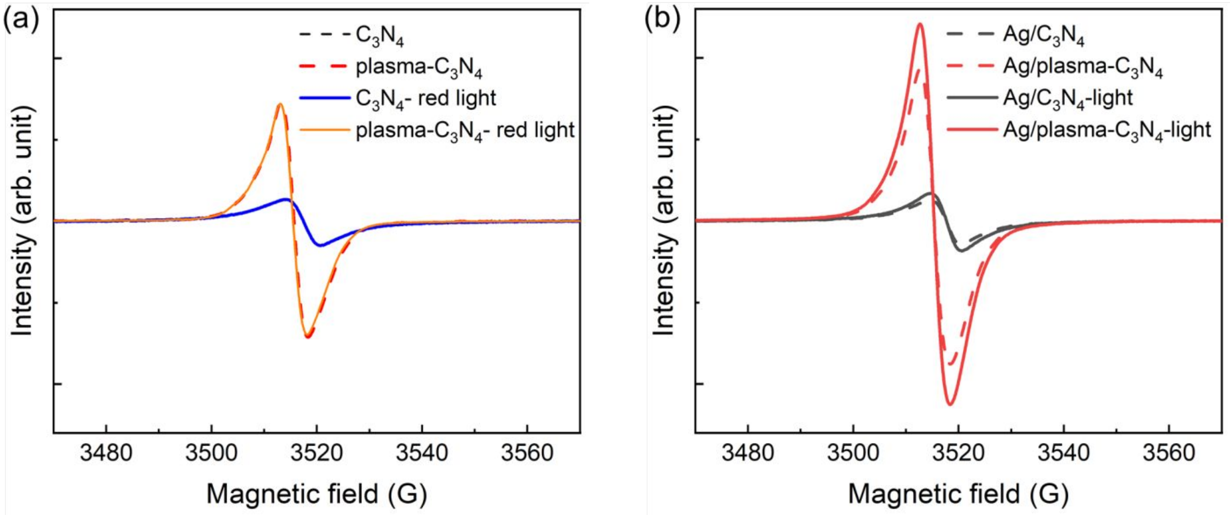

Figure S13 ESR spectra of (a) C_3N_4 and plasma-C_3N_4 measured in the dark (dashed lines) or under red light (λ_{ex} > 550 nm) irradiation (solid lines) and (b) Ag/C_3N_4 and Ag/plasma-C_3N_4 measured in the dark (dashed lines) or under UV-vis light (750 nm λ_{ex} > 360 nm) irradiation (solid lines). In Figure 12a, the ESR spectrum of C_3N_4 (black dashed line) is overlapped by that of C_3N_4 under red light irradiation (blue solid line). Single Lorentzian peak was observed at g = 2.0042 for Ag/C_3N_4 and at g = 2.0048 for Ag/plasma-C_3N_4, both of which are very similar to that of C_3N_4 and plasma-C_3N_4, respectively.

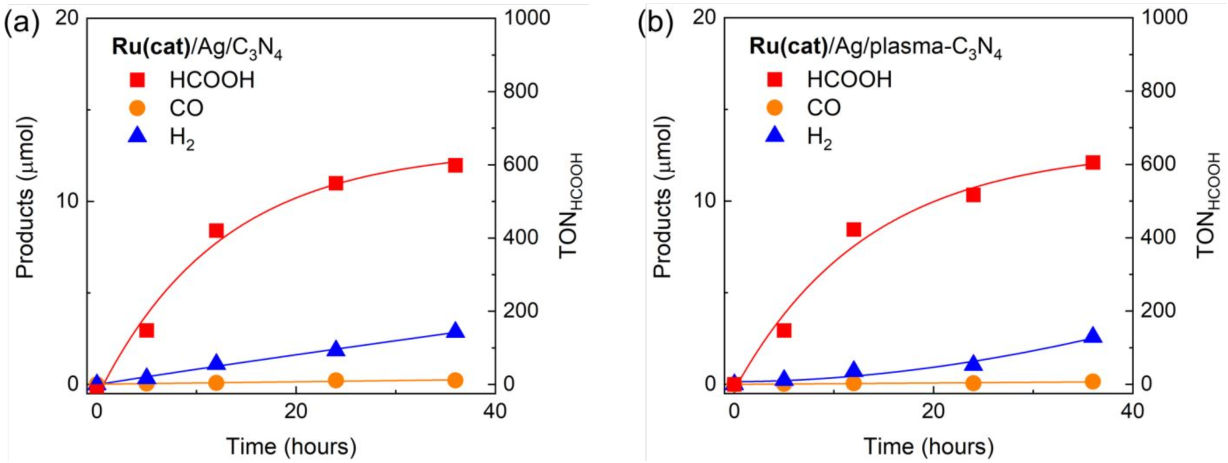

Figure S14 Time course of product amounts during the photocatalytic CO_2 reduction with (a) **Ru(cat)** (0.5 μmol g^{-1})/Ag (1.25wt%)/C_3N_4 (using as-C_3N_4) and (b) **Ru(cat)** (0.5 μmol g^{-1})/Ag (1.25wt%)/plasma-C_3N_4 (using plasma-C_3N_4). The photocatalytic reactions were performed in DMA/TEOA (4:1, v/v) solutions with stirring under CO_2 atmosphere through visible light irradiation using LEDs at λ_{ex}^{max} = 410 and 460 nm.

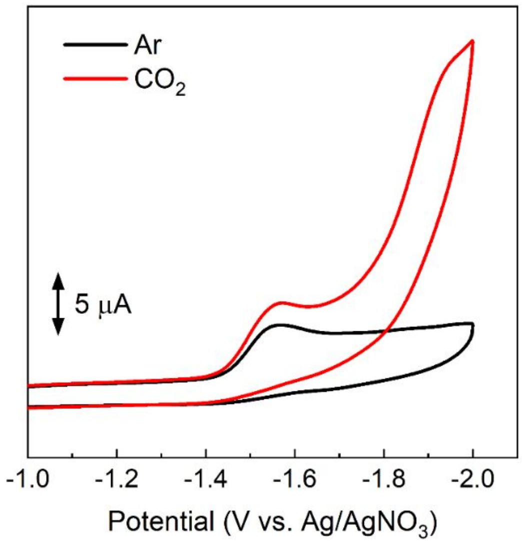

Figure S15 Cyclic voltammogram of 0.5 mM **Ru(cat)** in DMA/TEOA (v:v = 4:1) containing 0.1 M of Et_4NBF_4 as an electrolyte. WE: glassy carbon (3 mm), RE: $Ag/AgNO_3$, CE: Pt, Scan rate: 100 mV s^{-1}. The reduction potential of **Ru(cat)** was estimated from the peak potential of the first reduction wave measured in an Ar-bubbled solution. The increase in current measured in a CO_2-bubbled solution indicates the electrochemical reduction of CO_2.

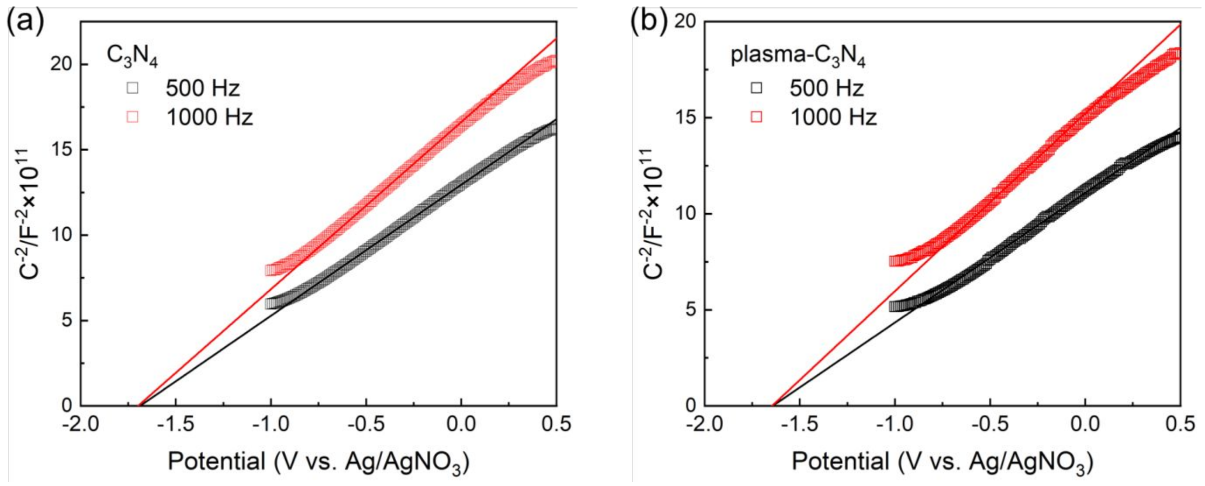

Figure S16 Mott–Schottky plots C_3N_4 (a) before and (b) after plasma surface modification, measured in DMA containing 0.1 M Et_4NBF_4 as an electrolyte. WE: FTO electrode on which C_3N_4 was deposited, RE: $Ag/AgNO_3$, CE: Pt. Flat band potentials of C_3N_4 before and after the plasma treatment were estimated to be −1.69 V and −1.64 V (vs $Ag/AgNO_3$), respectively. The conduction band potentials of the n-type semiconductor are empirically known to be located 0.1–0.3 V more negative than the flat-band potential,

depending on the conductivity. Because the conductivity of C_3N_4 was unclear, the difference between the flat-band potential and the conduction band minimum was assumed to be 0.2 V, which estimates the conduction band minimum of as- and plasma-C_3N_4 are −1.89 V and −1.84 V (vs Ag/$AgNO_3$), respectively.
